# MKRN3 inhibits puberty onset via interaction with IGF2BP1 and regulation of hypothalamic plasticity

**DOI:** 10.1172/jci.insight.164178

**Published:** 2023-04-24

**Authors:** Lydie Naulé, Alessandra Mancini, Sidney A. Pereira, Brandon M. Gassaway, John R. Lydeard, John C. Magnotto, Han Kyeol Kim, Joy Liang, Cynara Matos, Steven P. Gygi, Florian T. Merkle, Rona S. Carroll, Ana Paula Abreu, Ursula B. Kaiser

**Affiliations:** 1 Division of Endocrinology, Diabetes and Hypertension, Brigham and Women’s Hospital and Harvard Medical School, Boston, Massachusetts, USA.; 2 Department of Cell Biology, Harvard Medical School, Boston, Massachusetts, USA.; 3 Metabolic Research Laboratories and Medical Research Council Metabolic Diseases Unit, Wellcome Trust – Medical Research Council Institute of Metabolic Science and; 4 Wellcome Trust – Medical Research Council Cambridge Stem Cell Institute, University of Cambridge, Cambridge, United Kingdom.

**Keywords:** Endocrinology, Neuroscience, Mouse models, Neurodevelopment, Neuroendocrine regulation

## Abstract

Makorin ring finger protein 3 (MKRN3) was identified as an inhibitor of puberty initiation with the report of loss-of-function mutations in association with central precocious puberty. Consistent with this inhibitory role, a prepubertal decrease in *Mkrn3* expression was observed in the mouse hypothalamus. Here, we investigated the mechanisms of action of MKRN3 in the central regulation of puberty onset. We showed that *MKRN3* deletion in hypothalamic neurons derived from human induced pluripotent stem cells was associated with significant changes in expression of genes controlling hypothalamic development and plasticity. *Mkrn3* deletion in a mouse model led to early puberty onset in female mice. We found that *Mkrn3* deletion increased the number of dendritic spines in the arcuate nucleus but did not alter the morphology of GnRH neurons during postnatal development. In addition, we identified neurokinin B (NKB) as an Mkrn3 target. Using proteomics, we identified insulin-like growth factor 2 mRNA-binding protein 1 (IGF2BP1) as another target of MKRN3. Interactome analysis revealed that IGF2BP1 interacted with MKRN3, along with several members of the polyadenylate-binding protein family. Our data show that one of the mechanisms by which MKRN3 inhibits pubertal initiation is through regulation of prepubertal hypothalamic development and plasticity, as well as through effects on NKB and IGF2BP1.

## Graphical abstract

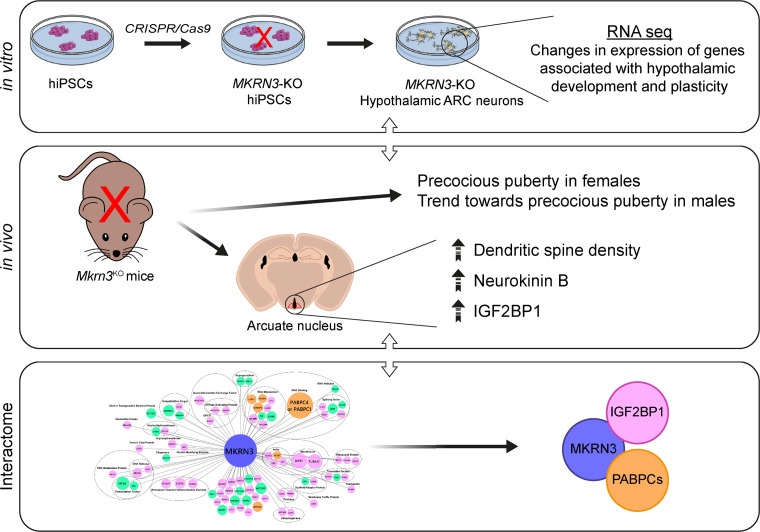

## Introduction

*MKRN3* (Makorin ring finger protein 3) was identified as an inhibitor of puberty initiation when loss-of-function mutations in *MKRN3* were first detected in patients with familial central precocious puberty (CPP) in 2013 (1). Subsequently, mutations in *MKRN3* have been identified in many additional patients with CPP and are the most common genetic cause of familial CPP (2, 3). *MKRN3* is located on human chromosome 15q11-q13 in the Prader-Willi syndrome critical region, where it is maternally imprinted and thus expressed only from the paternal allele (4, 5). In the rodent hypothalamus, *Mkrn3* expression is high from embryonic day 10.5 to the second week of postnatal development, then decreases sharply before puberty initiation (1, 6–8). The expression profile and identification of loss-of-function mutations in patients with early pubertal development support the hypothesis that MKRN3 acts as an inhibitor of GnRH secretion during the childhood quiescence of the hypothalamic-pituitary-gonadal (HPG) axis.

Puberty is a critical developmental period characterized by the acquisition of sexual maturity. Within the HPG axis, puberty is triggered by an increase in secretion of gonadotropin-releasing hormone (GnRH), which in turn stimulates the secretion of the pituitary gonadotropins necessary for activation of gonadal function. GnRH is secreted in a pulsatile manner during embryonic and neonatal development, followed by a period of quiescence during infancy, and subsequently a reactivation of its secretion at puberty (9). Early reactivation of pulsatile GnRH secretion results in CPP, defined in humans as pubertal onset before age 8 years in girls and before age 9 years in boys (10). In humans, the neuropeptides kisspeptin and neurokinin B (NKB) have been identified as major activators of pulsatile GnRH secretion, with loss-of-function mutations in *KISS1* (encoding kisspeptin) and *TAC3* (encoding NKB) genes associated with hypogonadotropic hypogonadism (HH) (11–15). In the mouse hypothalamus, GnRH neurons are localized primarily in the rostral preoptic area (rPOA) and send projections to the median eminence (ME). Kisspeptin neurons are distributed in 2 distinct regions, in the rostral periventricular area (RP3V), a subsection of the preoptic area (POA), and in the arcuate nucleus (ARC). Within the ARC, NKB is coexpressed with kisspeptin and is essential for coordination of GnRH pulses (16). Within the tachykinin family, neurokinin A and substance P, both encoded by *Tac1*, have also been described to regulate pubertal timing in rodents (17, 18).

In addition to significant changes in expression of these key GnRH regulators, the GnRH neuronal network undergoes a host of morphological and synaptic changes during postnatal/prepubertal hypothalamic development (19, 20). For example, morphological analyses revealed remodeling of the dendritic structure and spine density of GnRH neurons across postnatal and prepubertal development. Immature multipolar GnRH neurons undergo complex dendritic reorganization to achieve unipolar or bipolar arrangements typical of mature GnRH neurons (21). A postnatal increase in the number of spines has also been observed at the level of GnRH dendrites (21, 22). These developmental changes are essential for the proper maturation of the neural circuits governing the juvenile-adult transition.

Progress has been made in elucidating the mechanisms underlying the inhibitory action of MKRN3 on GnRH secretion. MKRN3 belongs to the makorin family of ubiquitin ligases (23). Its amino acid sequence contains a centrally located RING zinc finger motif (C3HC4), 2 amino-terminal C3H zinc finger motifs followed by a unique Cys-His configuration, and a final C3H motif. We and others have shown that MKRN3 binds to ubiquitin (6, 24). Based on its structure and its protein homology with MKRN1 and MKRN2, it has been suggested that MKRN3 may also possess RNA-binding activities. Thus, MKRN3 can potentially regulate both posttranscriptional RNA processing and posttranslational control of protein function (4, 23, 25). Several MKRN3 protein interactors have been identified in vitro, including neuronal pentraxin-1 (NPTX1), protein lin-28 homolog B (LIN28B), OTU domain-containing protein 4 (OTUD4), methyl-CpG-binding domain protein 3 (MBD3) and polyadenylate-binding proteins (PABPC1, PABPC3, PABPC4) (24, 26–28). However, additional studies to understand the molecular mechanisms of action of MKRN3 are still needed.

Recently, we found that *Mkrn3* is expressed in the mediobasal hypothalamus (MBH) and the POA and most notably in kisspeptin neurons (6). We also demonstrated that MKRN3 can selectively inhibit *KISS1* and *TAC3* promoter activity in vitro (6). These findings suggest an action of MKRN3 upstream of GnRH secretion via regulation of kisspeptin and NKB (6).

In the current study, we aimed to investigate further the role and mechanism of action of MKRN3 in the central regulation of puberty onset. First, using hypothalamic neurons derived from human induced pluripotent stem cells (hiPSCs), we showed that deletion of *MKRN3* was associated with significant changes in expression of genes controlling hypothalamic development and plasticity. We then characterized the pubertal phenotype of an *Mkrn3*-knockout mouse model and demonstrated that, similar to the human phenotype, *Mkrn3* deletion induced an advanced puberty onset in females and a trend toward an earlier age of puberty initiation in male mice. In addition, we showed that Mkrn3 regulates the density of dendritic spines in the ARC during postnatal development. Finally, we identified NKB and insulin-like growth factor 2 mRNA-binding protein 1 (IGF2BP1) as targets of the inhibitory action of Mkrn3 in the hypothalamus.

## Results

### Generation of wild-type and MKRN3-deficient hiPSC-derived hypothalamic neurons.

**Figure 1:**
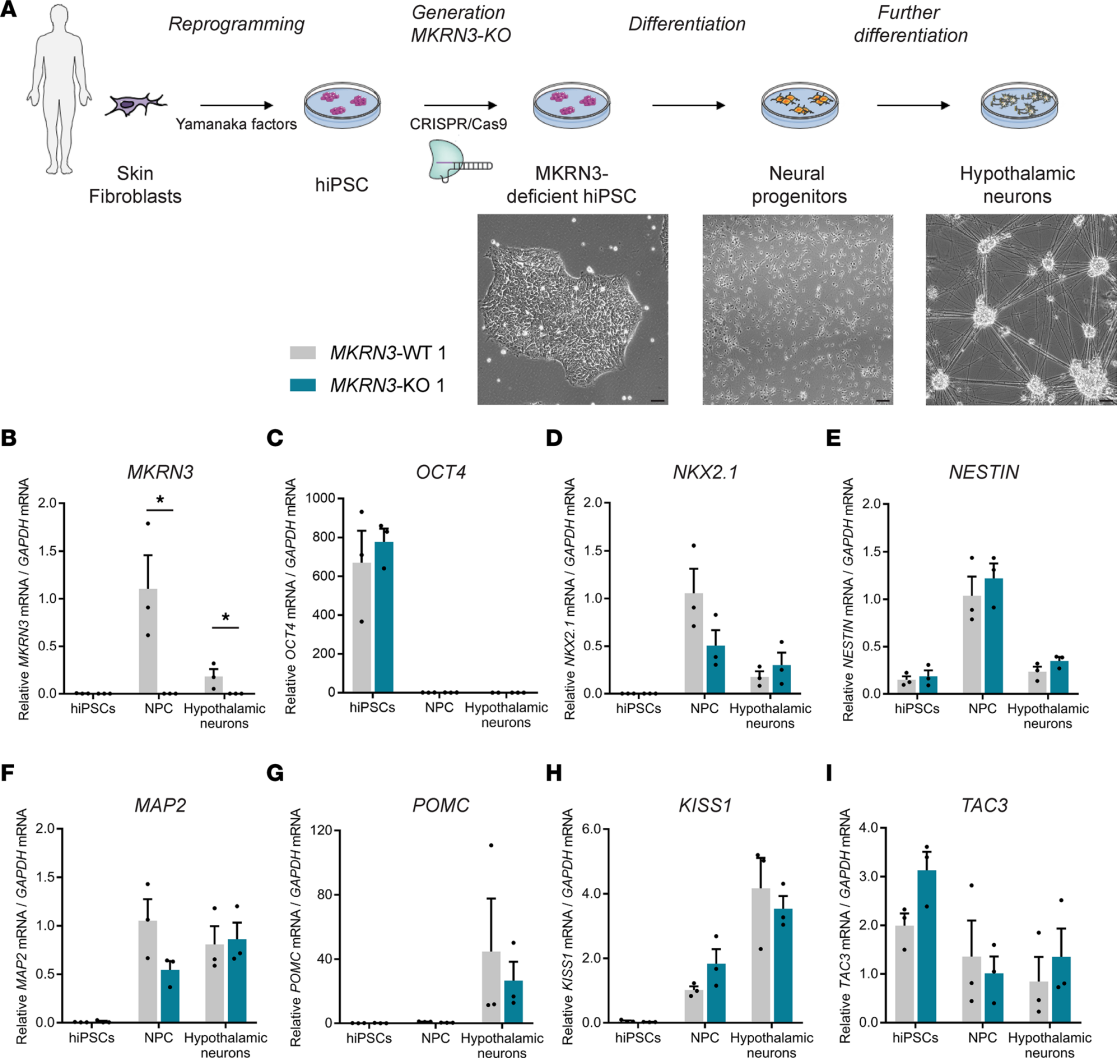
Generation of *MKRN3*-WT and *MKRN3*-knockout hiPSC-derived hypothalamic ARC neurons. (**A**) Schematic representation of the differentiation protocol for the generation of hypothalamic neurons from *MKRN3*-WT and *MKRN3*-KO hiPSCs, including live-cell imaging of hiPSCs before differentiation, neural progenitors (NPCs) on day 16 of differentiation, and hypothalamic ARC neurons on day 30 of differentiation. (**B**–**I**) *MKRN3* (**B**), *OCT4* (**C**), *NKX2.1* (**D**), *NESTIN* (**E**), *MAP2* (**F**), *POMC* (**G**), *KISS1* (**H**), and *TAC3* (**I**) mRNA levels (relative to levels in *MKRN3*-WT 1 NPCs) in hiPSCs, NPCs, and hypothalamic ARC neurons derived from the *MKRN3*-WT 1 and *MKRN3*-KO 1 clones (*n* = 3 differentiation protocols per group). Data are presented as the mean ± SEM. Statistics were performed using 2-way ANOVA, followed by Tukey’s post hoc test. *P < 0.01. KO, knockout; OCT4, octamer-binding transcription factor 4; NKX2.1, NK2 homeobox 1; NESTIN, Nestin; MAP2, microtubule associated protein 2; POMC, proopiomelanocortin.

To investigate the roles and mechanisms of action of MKRN3 within the human hypothalamus, we first differentiated hiPSC lines into hypothalamic neurons with many features and characteristics of ARC neurons, therefore referred to herein as hypothalamic ARC neurons (29, 30). *MKRN3* deletion was introduced into hiPSCs using CRISPR/Cas9 technology (Supplemental Figure 1, A and B; supplemental material available online with this article; https://doi.org/10.1172/jci.insight.164178DS1). Hypothalamic differentiation of 2 clones for both *MKRN3*-WT hiPSCs (*MKRN3*-WT 1, Figure 1, B–I; and *MKRN3*-WT 2, Supplemental Figure 1, C–J) and *MKRN3*-deficient hiPSCs (*MKRN3*-KO 1, Figure 1, B–I; and *MKRN3*-KO 2, Supplemental Figure 1, C–J) was performed using an established hypothalamic neuron differentiation protocol (29) (Figure 1A). Real-time quantitative PCR (RT-qPCR) analysis verified that *MKRN3* expression was absent in the *MKRN3*-KO cells (*F*_1,12_ = 12.80, *P* = 0.004) (Figure 1B and Supplemental Figure 1C). In *MKRN3*-WT cells, *MKRN3* expression was markedly induced by the differentiation protocol (Figure 1B and Supplemental Figure 1C). *MKRN3* mRNA was not detected in hiPSCs but was expressed in the WT neural progenitor cells (NPCs) (at day 16 of differentiation) and in the WT hypothalamic neurons (day 30) (*F*_2,12_ = 8.098, *P* = 0.006). The data showed that over the neural induction period, *OCT4*-expressing hiPSCs differentiated with high efficiency into *NKX2.1-* and *NESTIN*-expressing NPCs (*OCT4*: *F*_2,11_ = 58.32, *P* < 0.0001; *NKX2.1*: *F*_2,12_ = 17.17, *P* = 0.0003; *NESTIN*: *F*_2,12_ = 43.61, *P* < 0.0001) (Figure 1, C–E, and Supplemental Figure 1, D–F). In addition, expression of the neuronal marker *MAP2* verified the differentiation of the hiPSCs into neurons (*F*_2,12_ = 21.52, *P* = 0.0001) (Figure 1F and Supplemental Figure 1G). Furthermore, *POMC* and *KISS1*, 2 markers of the ARC, were induced during the hypothalamic differentiation protocol (*POMC*: *F*_2,12_ = 4.015, *P* = 0.046; *KISS1*: *F*_2,12_ = 35.54, *P* < 0.0001) (Figure 1, G and H, Supplemental Figure 1, H and I). *TAC3* was expressed in hiPSCs, NPCs, and hypothalamic ARC neurons (Figure 1I and Supplemental Figure 1J). No significant differences were observed in *NKX2.1* (*F*_1,12_ = 1.641, *P* = 0.224), *NESTIN* (*F*_1,12_ = 1.480, *P* = 0.247), and *MAP2* (*F*_1,12_ = 1.643, *P* = 0.224) expression between *MKRN3*-WT and *MKRN3*-KO hypothalamic ARC neurons. *POMC*, *KISS1*, and *TAC3* were also expressed at similar levels in both *MKRN3*-WT and *MKRN3*-KO hypothalamic neurons (*POMC*: *F*_1,12_ = 0.286, *P* = 0.603; *KISS1*: *F*_1,12_ = 0.021, *P* = 0.889; *TAC3*: *F*_1,12_ = 1.150, *P* = 0.305) (Figure 1, G–I, and Supplemental Figure 1, H–J). These results indicate that *MKRN3* expression is highly upregulated during the differentiation of hypothalamic ARC neurons yet not essential for this process.

### MKRN3 deletion in hiPSC-derived hypothalamic ARC neurons is associated with changes in expression of genes associated with hypothalamic development and plasticity.

**Figure 2:**
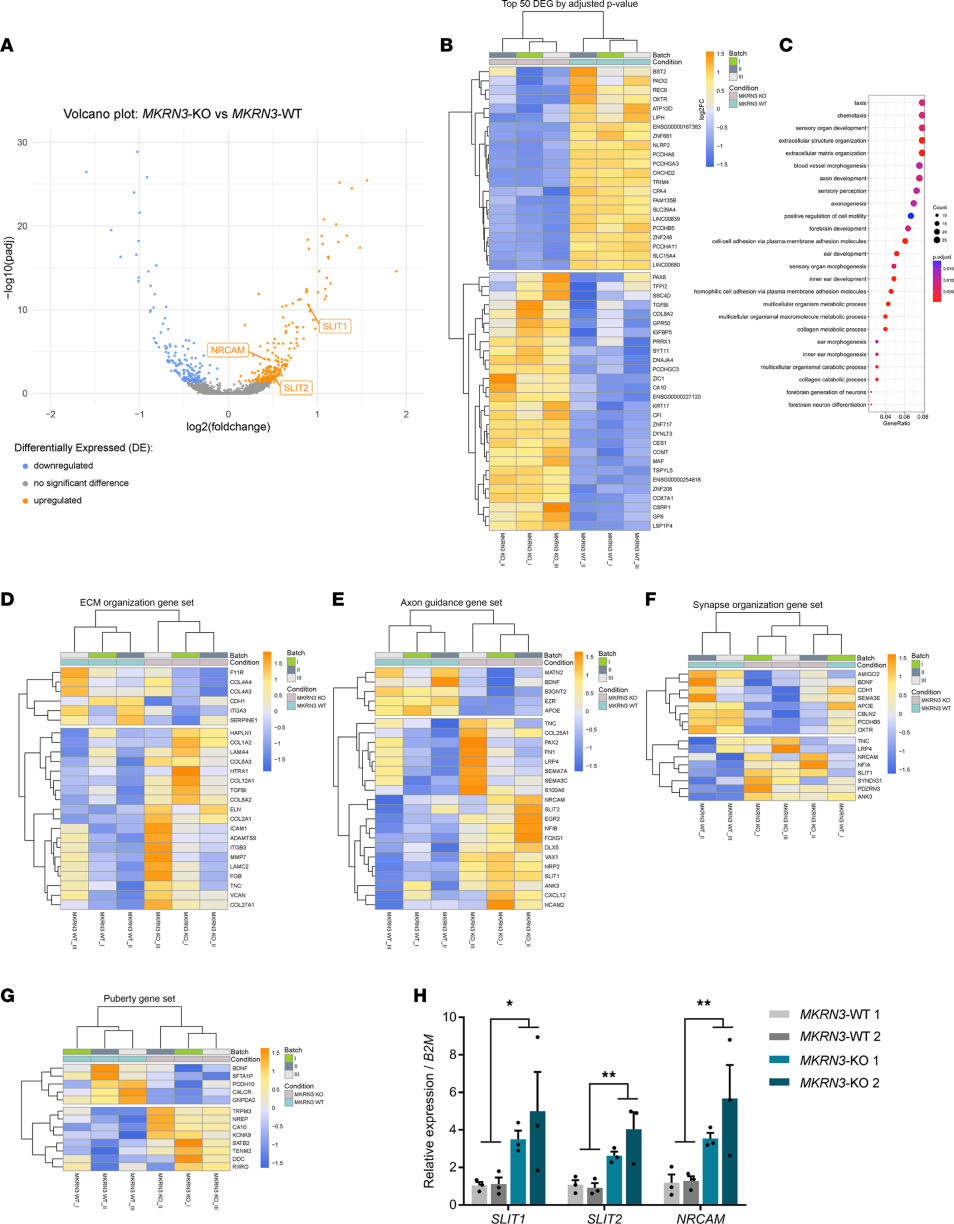
Comparison of transcriptomes of *MKRN3*-KO and *MKRN3*-WT hiPSC-derived hypothalamic ARC neurons reveals differences in expression of genes that control hypothalamic neuronal development and plasticity. (**A**) Volcano plot comparing Benjamini-Hochberg–adjusted (BH-adjusted) *P* values against fold-change, showing transcripts that are differentially expressed between *MKRN3*-KO and *MKRN3*-WT hypothalamic ARC neurons (genes downregulated in *MKRN3*-KO neurons in blue, genes upregulated in *MKRN3*-KO neurons in orange, and genes not significantly different in gray). The analysis was performed using a BH adjusted *P* value cutoff of 0.05 and a log_2_ fold-change ratio cutoff of 1. Three representative genes more highly expressed in *MKRN3*-KO are shown. *SLIT1*, slit guidance ligand 1; *SLIT2*, slit guidance ligand 2; *NRCAM*, neuronal cell adhesion molecule. (**B**) Heatmap of the top 50 genes that are the most differentially expressed in *MKRN*3-KO 1 compared with *MKRN3*-WT 1 hypothalamic neurons. Each column represents 1 differentiation protocol. (**C**) Dot plot of Gene Ontology (GO) terms enriched between *MKRN3*-WT and *MKRN3*-KO hypothalamic neurons. The size of the dots represents the number of genes in the GO term, and the color gradient indicates the adjusted *P* value, using the BH method. (**D**–**F**) Heatmaps of differentially expressed genes (DEGs) in 3 significantly enriched pathways: (**D**) extracellular matrix (ECM) organization, (**E**) axon guidance, and (**F**) synapse organization, in *MKRN3*-KO compared with *MKRN3*-WT hypothalamic neurons. (**G**) Heatmap for a subset of DEGs between *MKRN3*-WT and *MKRN3*-KO hypothalamic neurons that have been associated with age at menarche in GWAS. (**H**) Relative *SLIT1*, *SLIT2*, and *NRCAM* mRNA levels in *MKRN3*-WT 1, *MKRN3*-WT 2, *MKRN3*-KO 1, and *MKRN3*-KO 2 hypothalamic neurons (*n* = 3 differentiation protocols per group). Data are presented as the mean ± SEM values. Statistics were performed using 2-way ANOVA, followed by Tukey’s post hoc test. *P < 0.05, **P < 0.01.

To identify hypothalamic targets of MKRN3, we performed comparative transcriptome analysis by RNA sequencing (RNA-Seq) of *MKRN3*-WT and *MKRN3*-KO hypothalamic ARC neurons after 30 days of differentiation. We identified 404 differentially expressed genes (DEGs), including 176 genes upregulated and 228 genes downregulated in the *MKRN3*-KO hypothalamic neurons in comparison with *MKRN3*-WT, as illustrated in a volcano plot (Figure 2A). Analysis was performed using a BH-adjusted *P* value cutoff of 0.05 and a log fold-change ratio cutoff of 1. The top 50 DEGs are presented in Figure 2B. Gene ontology (GO) enrichment analysis revealed a list of 53 pathways that were differentially regulated (Figure 2C). Interestingly, the most enriched pathways included extracellular matrix organization, axon guidance, and synapse organization, which together control neuronal development and synaptic plasticity (Figure 2, D–F). In addition, we examined the transcript abundance of genes that have been associated with age at menarche. In humans, a recent genome-wide association study (GWAS) identified 389 independent regions associated with age at menarche (31, 32). Among them, 13 genes located in these loci are differentially expressed between *MKRN3-*KO and *MKRN3*-WT hypothalamic ARC neurons, including the paternally imprinted gene potassium two pore domain channel subfamily K member 9 (*KCNK9*) and the gene teneurin transmembrane protein 2 (*TENM2*), a protein involved in neural development (33, 34) (Figure 2G). RT-qPCR analysis verified the increases in expression of *SLIT1*, *SLIT2*, and *NRCAM*, 3 genes critical to the development of the hypothalamus, in *MKRN3*-KO hypothalamic ARC neurons compared with *MKRN3*-WT (*SLIT1*: *F*_1,8_ = 8.513, *P* = 0.019; *SLIT2*: *F*_1,8_ = 21.17, *P* = 0.002; *NRCAM*: *F*_1,8_ = 12.97, *P* = 0.007) (35–37) (Figure 2H). Taken together, these findings suggest that *MKRN3* plays an important role in the regulation of ARC development and plasticity. Puberty has been shown to be a critical time window for neuronal development and plasticity (20, 38). Thus, these observations suggest a potential role for MKRN3 in regulating these processes prior to and during puberty initiation.

### Mkrn3 deletion in mice induces early onset of puberty in female mice and a tendency toward early puberty in male mice.

**Figure 3:**
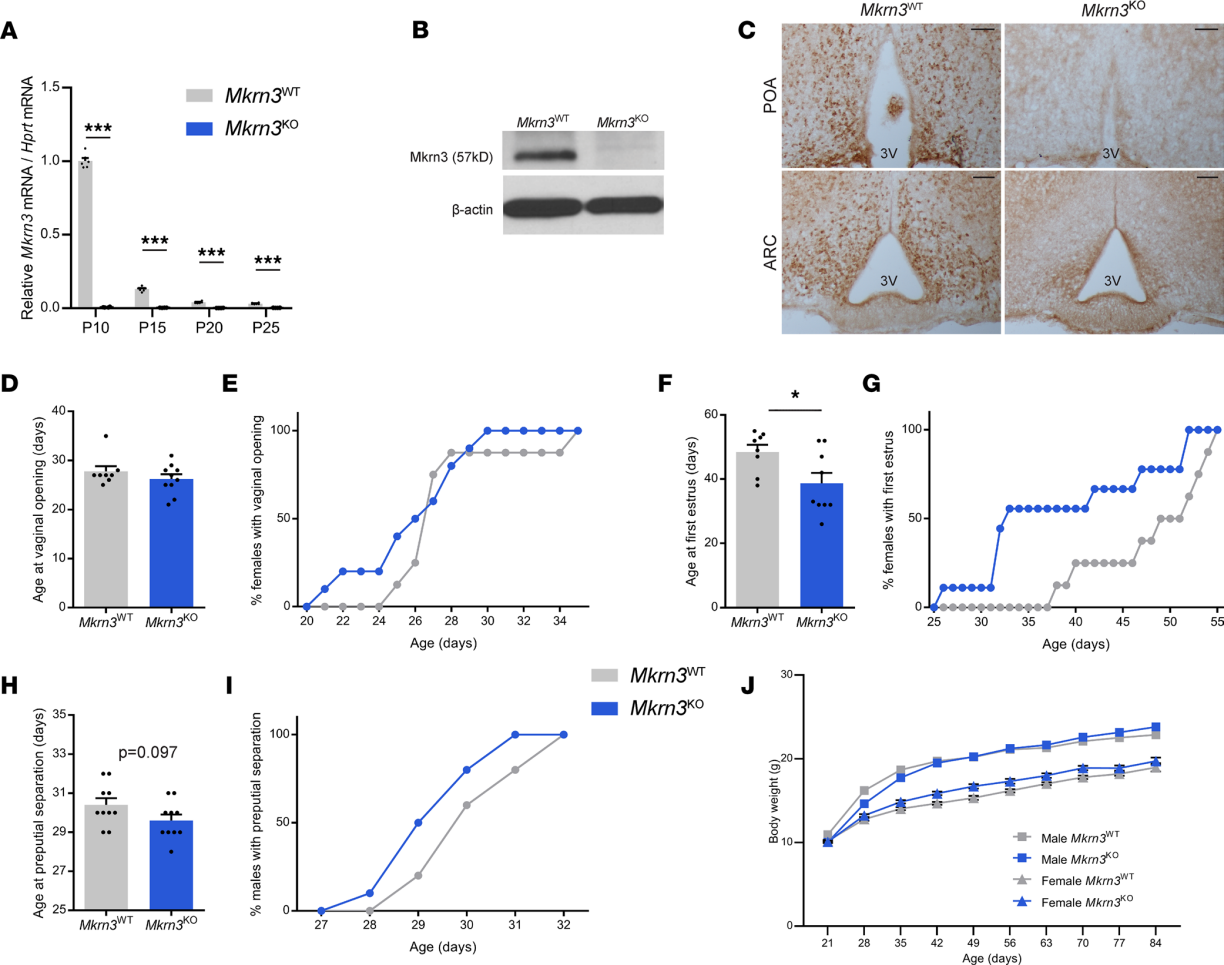
*Mkrn3* deletion in mice is associated with early onset of puberty in female mice and a tendency toward early puberty in male mice. (**A**) Relative *Mkrn3* mRNA levels in the ARC of *Mkrn3*^WT^ and *Mkrn3*^KO^ females across PND10, PND15, PND20, and PND25 (*n* = 6 per genotype and age). Statistics were performed using 2-way ANOVA, followed by Tukey’s post hoc test. (**B**) Representative Western blot autoradiographic image of Mkrn3 protein and β-actin in the MBH of *Mkrn3*^WT^ and *Mkrn3*^KO^ females at PND10. (**C**) Representative immunohistochemistry images of Mkrn3 protein in the POA and the arcuate nucleus (ARC) of *Mkrn3*^WT^ and *Mkrn3*^KO^ females at PND10. Scale bar = 100 μm. 3V, third ventricle. (**D**–**G**) Age at vaginal opening (**D**) and cumulative percentage of female mice exhibiting vaginal opening (**E**), and age at first estrus (**F**) and cumulative percentage of female mice with first estrus (**G**), in *Mkrn3*^WT^ (*n* = 8) and *Mkrn3*^KO^ (*n* = 10) females. (**H**) Age at preputial separation and (**I**) cumulative percentage of male mice exhibiting preputial separation in *Mkrn3*^WT^ (*n* = 10) and *Mkrn3*^KO^ (*n* = 11) males. Statistics were performed using unpaired 2-tailed *t* test. (**J**) Body weight of *Mkrn3*^WT^ (*n* = 11) and *Mkrn3*^KO^ (*n* = 15) male and *Mkrn3*^WT^ (*n* = 8) and *Mkrn3*^KO^ (*n* = 10) female mice measured weekly from weaning (PND21) to adulthood (PND84). Statistics were performed using 2-way ANOVA, followed by Tukey’s post hoc test. Data are presented as the mean ± SEM values. *P < 0.05, ***P < 0.0001 of *Mkrn3*^KO^ compared with *Mkrn3*^WT^.

To further investigate the role of Mkrn3 in the hypothalamic regulation of the reproductive axis and explore its mechanisms of action, we generated an *Mkrn3*-deficient mouse model by homologous recombination. We first confirmed that *Mkrn3* is maternally imprinted and expressed only from the paternally inherited allele in our mouse model. Our analysis validated that when the *Mkrn3* deletion originated from the paternal allele (*Mkrn3*^+/–^), there was no *Mkrn3* gene expression in the hypothalamus (*t*_4_ = 5.191, *P* = 0.0066) (Supplemental Figure 2A), whereas when the deletion originated from the maternally inherited allele (*Mkrn3*^–/+^), the expression was similar to the *Mkrn3*-WT (*Mkrn3*^+/+^) mice (*t*_5_ = 1.368, *P* = 0.230) (Supplemental Figure 2B). Thus, subsequent analyses compared *Mkrn3*^+/–^ (from here onward referred to as *Mkrn3*^KO^) and *Mkrn3*^+/+^ (referred to as *Mkrn3*^WT^) littermates. As previously reported, RT-qPCR analysis verified that *Mkrn3* mRNA levels were high in the hypothalamic ARC during early postnatal development, with a sharp decline starting at postnatal day (PND) 15, and remained low through PND25 (*F*_3,38_ = 1,599, *P* < 0.0001) (1, 6, 7). *Mkrn3* expression in the ARC was absent in the *Mkrn3*^KO^ littermates at all ages (*F*_1,38_ = 2,827, *P* < 0.0001) (Figure 3A). By Western blot (Figure 3B) and immunohistochemistry (Figure 3C), we also verified that Mkrn3 protein was undetectable in the ARC of *Mkrn3*^KO^ mice compared with *Mkrn3*^WT^ at PND10.

Pubertal phenotyping analyses showed no difference in the day of vaginal opening in *Mkrn3*^KO^ compared with *Mkrn3*^WT^ females (*t*_16_ = 1.063, *P* = 0.303, Figure 3, D and E) but a significant advance in the day of first estrus (*t*_15_ = 2.416, *P* = 0.029, Figure 3F). Detailed analysis of the cumulative percentage of females exhibiting first estrus showed a marked leftward shift in *Mkrn3*^KO^ compared with *Mkrn3*^WT^ animals (Figure 3G). In males, a trend toward earlier age of preputial separation was observed in *Mkrn3*^KO^ compared with *Mkrn3*^WT^ animals, though it did not reach statistical significance (*t*_18_ = 1.750, *P* = 0.097, Figure 3, H and I). These changes in pubertal timing were not associated with any differences in body weight between *Mkrn3*^KO^ and *Mkrn3*^WT^ animals (females: *F*_1,16_ = 0.878, *P* = 0.3626; males: *F*_1,24_ = 0.037, *P* = 0.849) (Figure 3J). No subsequent differences in adult female estrous cyclicity or in male or female fertility were observed in adulthood between *Mkrn3*^WT^ and *Mkrn3*^KO^ mice (Supplemental Figure 3 and Supplemental Table 1).

### Hypothalamic Kiss1, Tac3, Tac1, and Gnrh1 expression is not different in Mkrn3^KO^ compared to Mkrn3^WT^ mice.

**Figure 4:**
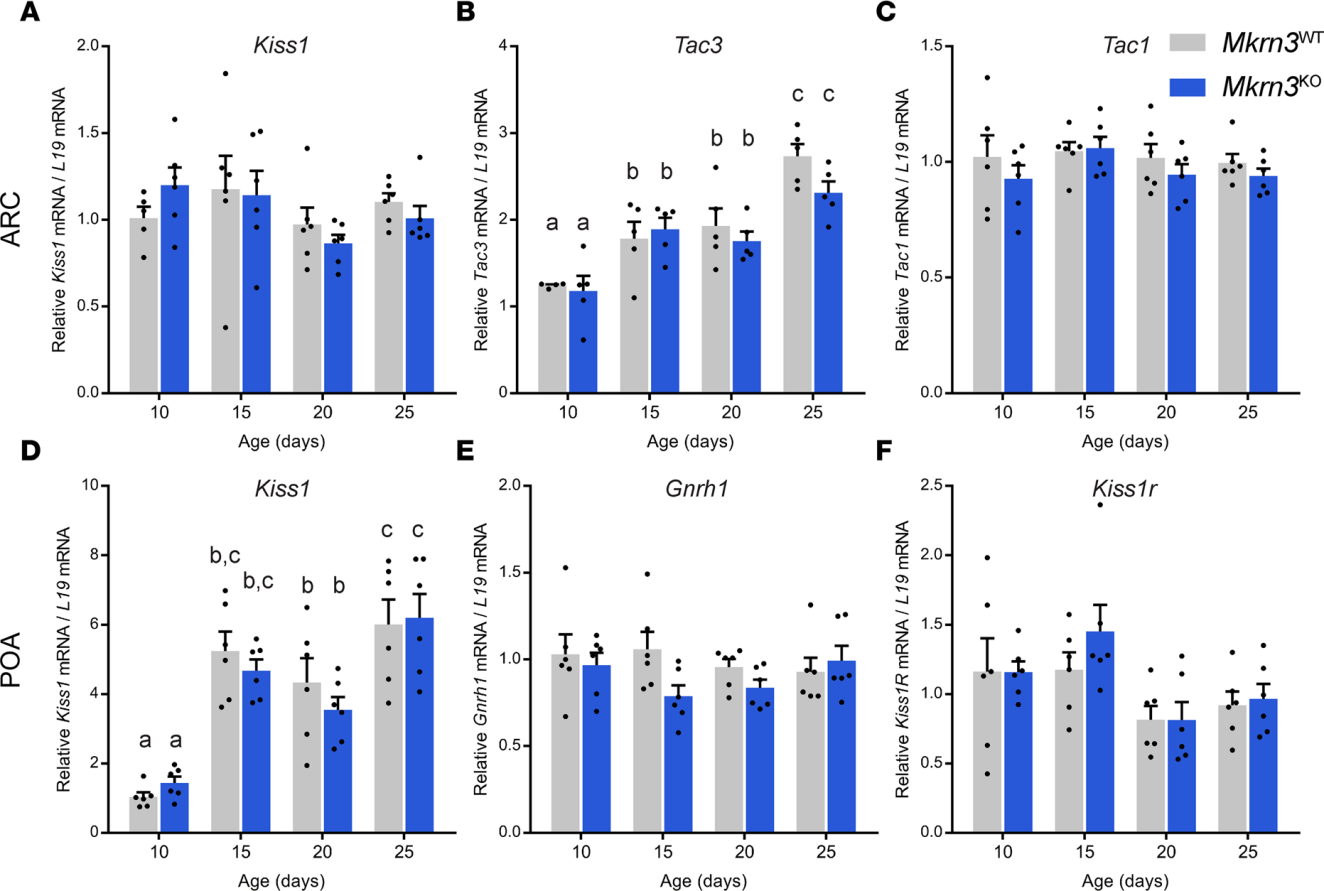
*Mkrn3* deletion in mice does not affect postnatal hypothalamic expression of *Kiss1*, *Tac3*, *Tac1*, *Gnrh1*, or *Kiss1r*. (**A**–**C**) Relative mRNA levels of (**A**) *Kiss1*, (**B**) *Tac3*, and (**C**) *Tac1* in the ARC of *Mkrn3*^WT^ and *Mkrn3*^KO^ females across postnatal development (PND10, PND15, PND20, and PND25) (*n* = 6 per genotype and per age). (**D**–**F**) Relative mRNA levels of (**D**) *Kiss1*, (**E**) *Gnrh1*, and (**F**) *Kiss1r* in the POA of *Mkrn3*^WT^ and *Mkrn3*^KO^ females at PND10, PND15, PND20, and PND25 (*n* = 6 per genotype and per age). Statistics were performed using 2-way ANOVA, followed by Tukey’s post hoc test. Data are presented as the mean ± SEM. Bars with different letters are significantly different from one another (*P* < 0.05).

To determine the central mechanisms of action of Mkrn3 in regulating puberty initiation, we examined the expression of hypothalamic genes known to regulate the reproductive axis. Similar to our studies in hiPSC-derived hypothalamic ARC neurons, we measured *Kiss1* and *Tac3* mRNA levels in the ARC of *Mkrn3*^WT^ and *Mkrn3*^KO^ females at different ages across postnatal development (PND10, PND15, PND20, and PND25). We also measured mRNA levels of *Tac1* in the ARC. We further measured mRNA levels of *Kiss1*, *Gnrh1* (which encodes GnRH), and *Kiss1r* (which encodes the kisspeptin receptor) in the POA. As previously reported (39–41), we found a significant effect of age on *Tac3* expression in the ARC (*F*_3,31_ = 23.90, *P* < 0.0001, Figure 4B) and on *Kiss1* expression in the POA (*F*_3,40_ = 33.44, *P* < 0.0001, Figure 4D). However, no genotype differences were found in *Kiss1* (*F*_1,39_ = 0.026, *P* = 0.874), *Tac3* (*F*_3,31_ = 1.637, *P* = 0.210), or *Tac1* (*F*_1,40_ = 1.834, *P* = 0.183) mRNA levels in the ARC (Figure 4, A–C), or in *Kiss1* (*F*_1,40_ = 0.277, *P* = 0.601), *Gnrh1* (*F*_1,40_ = 2.999, *P* = 0.091), or *Kiss1r* (*F*_1,40_ = 0.603, *P* = 0.442) mRNA levels in the POA (Figure 4, D–F), between *Mkrn3*^WT^ and *Mkrn3*^KO^ mice, at any of the postnatal ages tested.

### NKB protein levels are increased in the ARC of Mkrn3^KO^ mice at PND25, compared with Mkrn3^WT^ mice.

**Figure 5:**
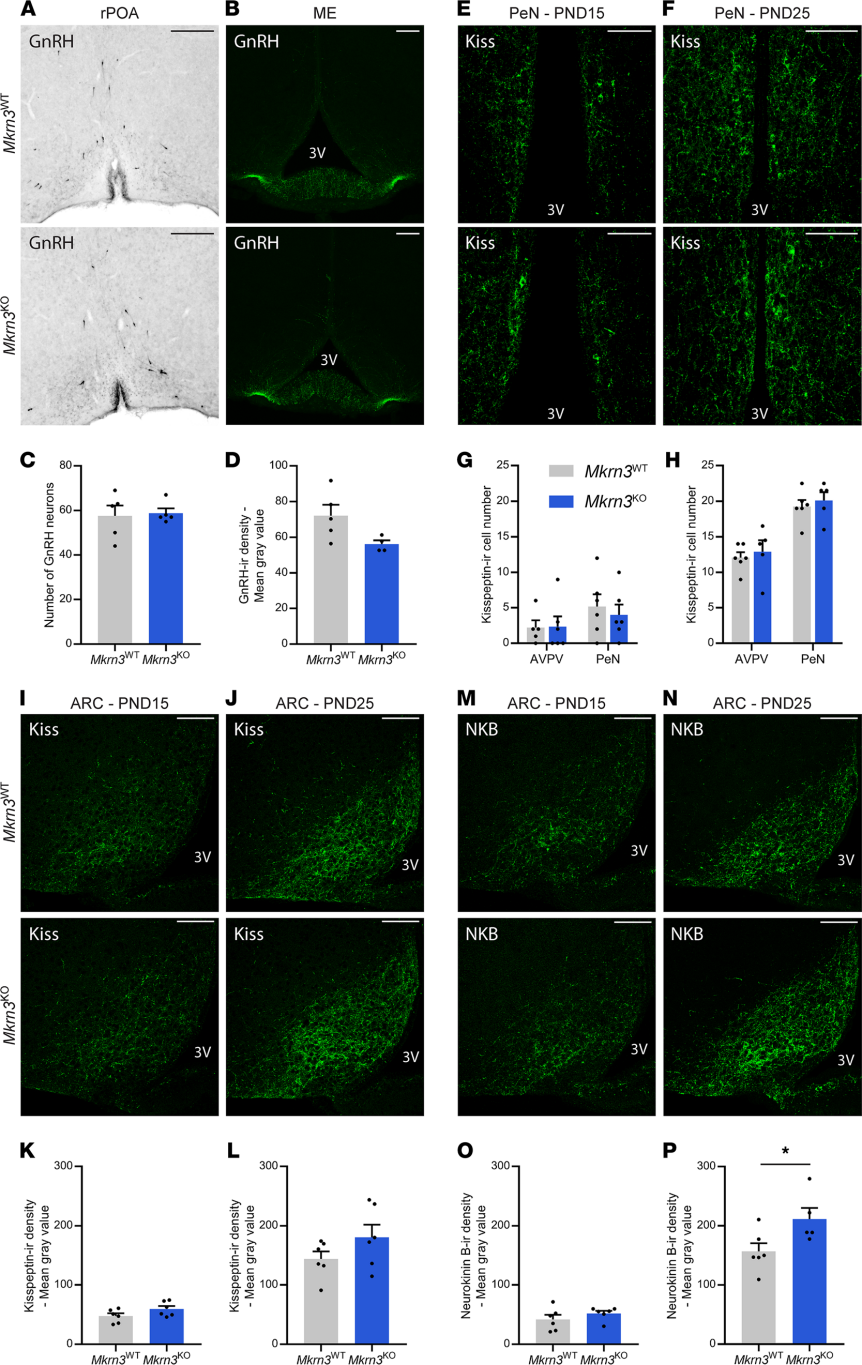
*Mkrn3* deletion in mice increases NKB protein levels in the ARC. (**A**–**D**) Representative images of (**A**) GnRH-immunoreactive (GnRH-ir) neurons in the rPOA and (**B**) fiber immunoreactivity in the ME of *Mkrn3*^WT^ and *Mkrn3*^KO^ females at PND10. (**C**) Quantification of the number of GnRH-ir neurons in the rPOA and (**D**) mean density of GnRH immunoreactivity in the ME (*n* = 5 per genotype). (**E**–**H**) Representative images of kisspeptin immunoreactivity in the periventricular nuclei (PeN) of the RP3V of *Mkrn3*^WT^ and *Mkrn3*^KO^ females at (**E**) PND15 and (**F**) PND25. Quantification of the number of kisspeptin-ir cells in the anteroventral periventricular (AVPV) area and the PeN at (**G**) PND15 and (**H**) PND25 (*n* = 5–6 per age and per genotype). (**I**–**L**) Representative images of kisspeptin immunoreactivity in the ARC of *Mkrn3*^WT^ and *Mkrn3*^KO^ females at (**I**) PND15 and (**J**) PND25. Quantification of the mean density of kisspeptin immunoreactivity in the ARC at (**K**) PND15 and (**L**) PND25 (*n* = 6 per age and genotype). (**M**–**P**) Representative images of NKB immunoreactivity in the ARC of *Mkrn3*^WT^ and *Mkrn3*^KO^ females at (**M**) PND15 and (**N**) PND25. Quantification of the mean density of NKB immunoreactivity in the ARC at (**O**) PND15 and (**P**) PND25 (*n* = 5–6 per age and genotype). Scale bar = 100 μm. Statistics were performed using unpaired 2-tailed *t* test. Data are presented as the mean ± SEM values. *P < 0.05 compared with *Mkrn3*^WT^.

Mkrn3 is an E3 ubiquitin ligase, a category of enzymes that mediate the transfer of ubiquitin from an E2 ubiquitin-conjugating enzyme to target protein substrates (6, 24). Ubiquitination can lead to a variety of effects on the protein substrate, ranging from proteasome-dependent proteolysis to modulation of protein function and/or localization (42, 43). To determine whether Mkrn3 could act at the protein level, we assessed the effect of *Mkrn3* deletion on levels of GnRH, kisspeptin, and NKB protein by immunohistochemistry. There was no significant difference in the number of GnRH neurons in the rPOA (*t*_8_ = 0.238, *P* = 0.818, Figure 5, A and C) or in the density of GnRH immunoreactivity in the ME (*t*_7_ = 2.238, *P* = 0.06, Figure 5, B and D) between *Mkrn3*^WT^ and *Mkrn3*^KO^ females at PND10. We also analyzed the number of kisspeptin-immunoreactive (kisspeptin-ir) cells in the POA, specifically in the anteroventral periventricular area (AVPV) and the preoptic periventricular nuclei (PeN), commonly referred to as the RP3V (Figure 5, E–H), as well as the mean density of kisspeptin immunoreactivity in the ARC (Figure 5, I–L), in female mice at PND15 and PND25. No significant changes in kisspeptin immunoreactivity between *Mkrn3*^WT^ and *Mkrn3*^KO^ animals were found in the RP3V (PND15, AVPV: *t*_9_ = 0.073, *P* = 0.944, PeN: *t*_10_ = 0.52, *P* = 0.614; PND25, AVPV: *t*_9_ = 0.487, *P* = 0.638, PeN: *t*_9_ = 0.578, *P* = 0.577) or in the ARC (PND15: *t*_10_ = 1.757, *P* = 0.109; PND25: *t*_10_ = 1.483, *P* = 0.169). Interestingly, while there were no differences in NKB immunoreactivity in the ARC identified at PND15 (*t*_10_ = 1.114, *P* = 0.291) (Figure 5, M and O), the mean density of NKB immunoreactivity in the ARC at PND25 was significantly higher in *Mkrn3*^KO^ than in *Mkrn3*^WT^ females (*t*_9_ = 2.399, *P* = 0.04, Figure 5, N and P). Thus, these data indicate that Mkrn3 is involved in regulation of NKB protein levels in the ARC.

### Mkrn3 deletion increases the number of dendritic spines in the ARC during postnatal development.

**Figure 6:**
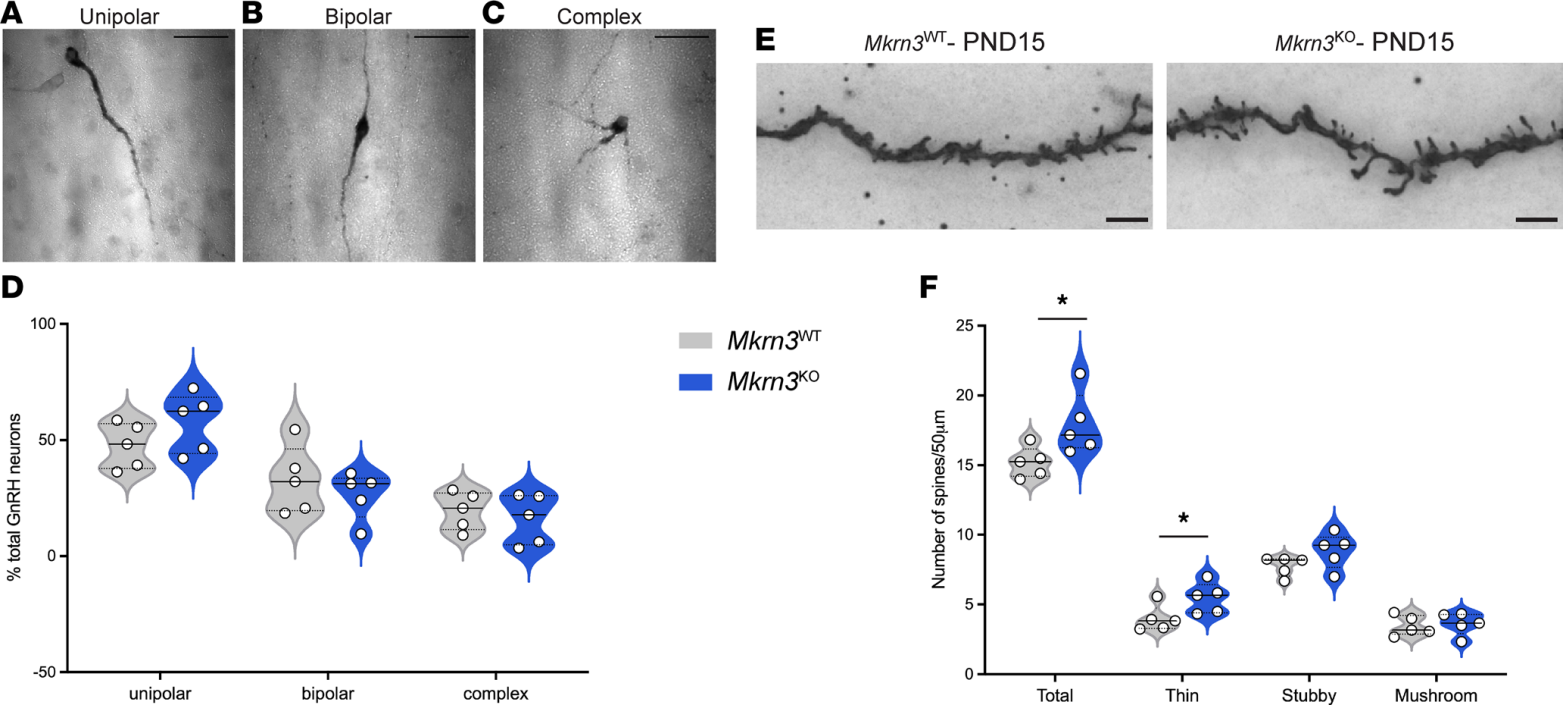
*Mkrn3* deletion does not alter GnRH neuron morphology but increases spine density in the ARC. (**A**–**C**) Representative images of GnRH neurons classified into (**A**) unipolar, (**B**) bipolar, and (**C**) complex dendritic morphology. Scale bar = 50 μm. (**D**) Quantification of the percentage of GnRH neurons in the rPOA with unipolar, bipolar, and complex morphology in *Mkrn3*^WT^ and *Mkrn3*^KO^ females at PND15 (*n* = 5 per genotype). Statistics were performed using 2-way ANOVA, followed by Tukey’s post hoc test. Data are presented as median and distribution of the data and probability density (violin plot). (**E**) Representative images of Golgi-Cox–impregnated neurons in the ARC of *Mkrn3*^WT^ and *Mkrn3*^KO^ females at PND15. Scale bar = 5 μm. (**F**) Quantification of the number of dendritic spines/50 μm in ARC neurons of *Mkrn3*^WT^ and *Mkrn3*^KO^ females at PND15 (*n* = 5 per genotype), classified as thin, stubby, or mushroom according to their morphology. Statistical analysis was performed using unpaired 2-tailed *t* tests. Data are presented as median and distribution of the data and probability density (violin plot). *P < 0.05 compared with *Mkrn3*^WT^.

Puberty is a period of intense hypothalamic structural and functional changes that allow the maturation of GnRH neurons and their neural network. Indeed, it has been shown that the GnRH neuron itself changes morphology across pubertal development, being significantly more complex before puberty compared with postpubertally (21). To investigate if Mkrn3 influences the development and plasticity of the hypothalamus before puberty initiation, we first analyzed the morphology of the GnRH neurons in the rPOA of PND15 female mice. As previously reported, GnRH neurons can be separated into unipolar, bipolar, and complex dendritic morphology (21). Immunohistochemistry for GnRH followed by confocal microscopy analyses did not show a significant difference in the morphology of GnRH neurons between *Mkrn3*^WT^ and *Mkrn3*^KO^ mice (*F*_1,24_ = 2.637 × 10^–8^, *P* = 0.999) (Figure 6, A–D). Subsequently, we assessed the number of dendritic spines present on neurons in the ARC using Golgi-Cox staining (Figure 6, E and F). Dendritic spines were classified as thin, stubby, or mushroom according to their morphology, from the least mature to the most mature spines, respectively (44). Interestingly, a significant increase of the total number of spines (*t*_8_ = 2.460, *P* = 0.039), and particularly in thin spines (*t*_8_ = 2.305, *P* = 0.050), was observed in *Mkrn3*^KO^ compared with *Mkrn3*^WT^ females at PND15 (Figure 6F). These results indicate that Mkrn3 participates in the regulation of dendritic spine morphology in hypothalamic neurons in the ARC, suggesting a role in postnatal plasticity of the ARC prior to puberty. These results are consistent with the results of the GO term analysis performed on the transcriptomes of *MKRN3*-WT and *MKRN3*-KO hiPSC-derived hypothalamic ARC neurons, which highlighted changes in the expression of genes that control neuronal development and synaptic plasticity (Figure 2).

### Mkrn3 reduces Igf2bp1 protein levels in the ARC during postnatal development.

**Figure 7:**
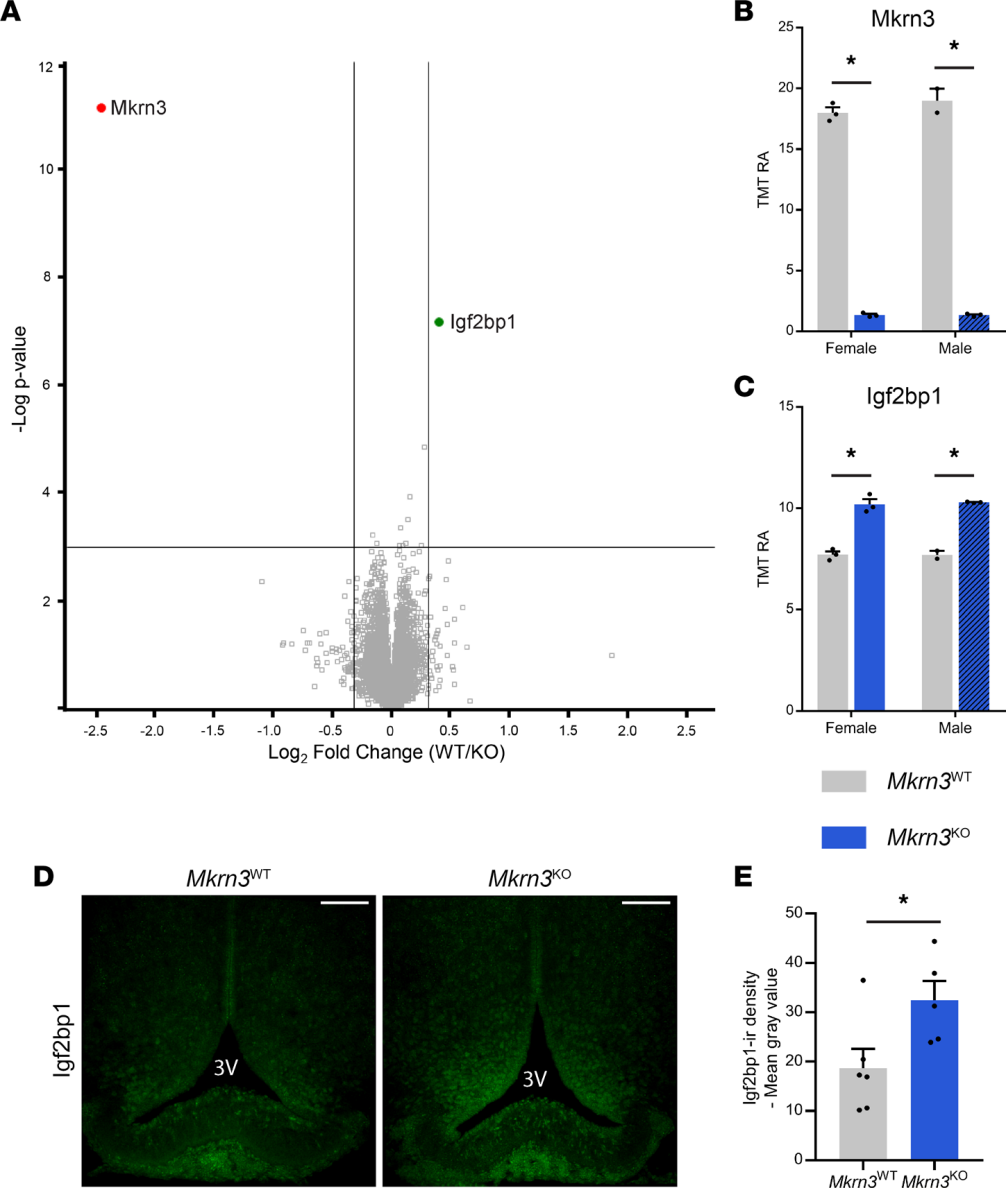
*Mkrn3* deletion increases Igf2bp1 protein levels in the ARC. (**A**) Volcano plot illustrating the significant changes in abundance of detected peptides in *Mkrn3*^KO^ compared with *Mkrn3*^WT^ mice in the ARC at PND15. Vertical lines represent a 25% increase in fold-change; horizontal line represents an FDR of 0.01 for *Mkrn3*^WT^ (*n* = 2) and *Mkrn3*^KO^ (*n* = 3) male and *Mkrn3*^WT^ (*n* = 3) and *Mkrn3*^KO^ (*n* = 3) female mice. (**B** and **C**) Relative TMT signal-to-noise levels for (**B**) Mkrn3 and (**C**) Igf2bp1 proteins in the ARC of PND15 *Mkrn3*^WT^ (*n* = 2) and *Mkrn3*^KO^ (*n* = 3) male and *Mkrn3*^WT^ (*n* = 3) and *Mkrn3*^KO^ (*n* = 3) female mice. TMT RA, tandem mass tag relative abundance. (**D**) Representative images of Igf2bp1 immunoreactivity in the ARC of *Mkrn3*^WT^ and *Mkrn3*^KO^ females at PND15. (**E**) Quantification of the mean density of Igf2bp1 immunoreactivity in the ARC of *Mkrn3*^WT^ (*n* = 6) and *Mkrn3*^KO^ (*n* = 5) females at PND15. Scale bar = 100 μm. Statistics were performed using unpaired 2-tailed *t* test. Data are presented as mean ± SEM. *P < 0.05 compared with *Mkrn3*^WT^ mice.

The ARC is the main region involved in the pubertal activation of pulsatile GnRH secretion (19). To further investigate possible targets of Mkrn3 action and to compare the data with our findings using hypothalamic ARC cells derived from hiPSCs, we again used an unbiased approach, in this case to compare the proteomes of the ARC of *Mkrn3*^WT^ and *Mkrn3*^KO^ animals at PND15, via Tandem Mass Tagging (TMT) (45). The TMT11-plex analysis quantified a total of 9,490 proteins (Supplemental Table 3). Among them, as expected, Mkrn3 was significantly decreased in the *Mkrn3*^KO^ animals, compared with *Mkrn3*^WT^ mice (Figure 7, A and B). Strikingly, Igf2bp1 showed the most significant increase in abundance in *Mkrn3*^KO^ animals, compared with *Mkrn3*^WT^ mice (Figure 7, A and C). This increase was verified by immunofluorescence analysis of Igf2bp1 protein levels in the ARC (Figure 7, D and E). Indeed, the mean density of Igf2bp1-ir cells in the ARC showed a significant increase in *Mkrn3*^KO^ females, compared with *Mkrn3*^WT^ female mice, at PND15 (*t*_9_ = 2.458, *P* = 0.036, Figure 7, D and E). Thus, these data suggest that Igf2bp1 is a target of Mkrn3 action.

### MKRN3 interacts with IGF2BP1 and PABPs.

**Figure 8:**
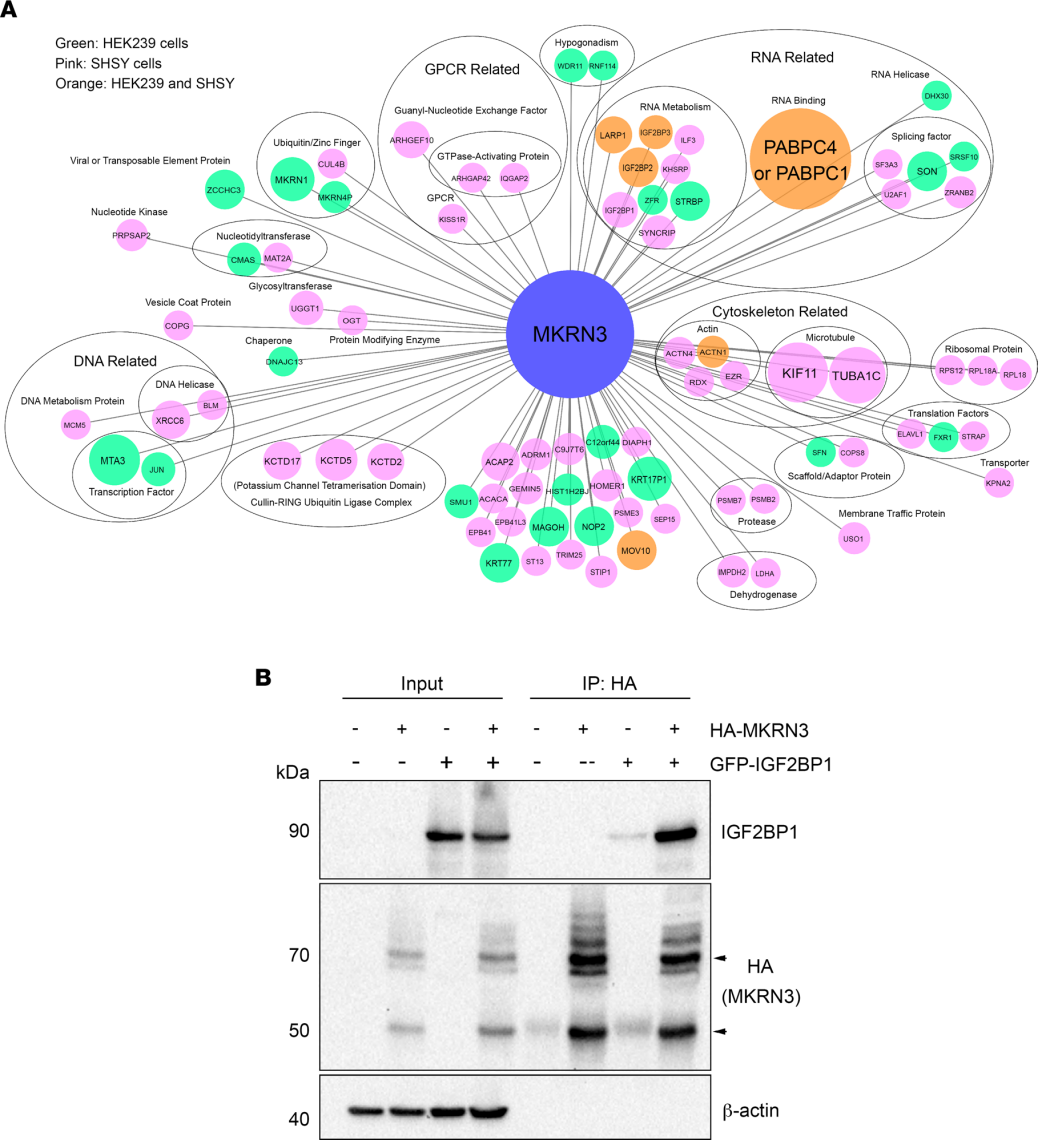
MKRN3 interacts with IGF2BP1. (**A**) Interactome map of key protein interactions with MKRN3. The network includes MKRN3 detected by different purification methods. Circle sizes indicate the CompPASS interaction score. Green circles indicate interaction identified in HEK293 cells, pink circles in SH-SY5Y cells, and orange circles in both cell lines. The oval nodes represent different clusters of prey proteins. (**B**) Co-IP analysis of MKRN3 and IGF2BP1 interaction. HEK293T cells were transiently transfected with HA-MKRN3, GFP-IGF2BP1, or both. Lysates were immunoprecipitated using anti-HA antibody. Both input and co-IP fractions were immunoblotted using anti-IGF2BP1 or anti-HA antibodies. The immunoblot demonstrates that IGF2BP1 is co-immunoprecipitated by anti-HA antibodies when coexpressed with HA-MKRN3.

As an E3 ubiquitin ligase, MKRN3 potentially acts through direct interaction with other proteins. Indeed, ubiquitination of proteins involves the participation of several enzymes and substrates (43). To build on our transcriptomic and proteomic data and to identify proteins that interact with MKRN3, we performed immunoprecipitation (IP) followed by mass spectrometry (MS) of proteins from 2 different cell types, HEK293 cells and human neuronally derived SH-SY5Y cells, transfected with N- and C-terminal HA-tagged MKRN3, with or without treatment with bortezomib, a proteasome inhibitor. After IP and parallel MS, we used a validated software tool, the Comparative Proteomic Analysis Software Suite (CompPASS), to filter contaminants and identify high-confidence candidate interacting proteins (HCIPs) (46). We identified 85 HCIPs that interacted with MKRN3, which included proteins implicated in RNA- and DNA-related processes, cytoskeleton function, ubiquitination machinery, and other functions (Figure 8A and Supplemental Table 4). Strikingly, IGF2BP1, 2, and 3 were among the strongest HCIPs identified. PABPC1 and 4, additional mRNA-binding proteins implicated in gene regulation and known targets of IGF2BP1, had the highest scores for interaction with MKRN3 in all conditions (47).

The interaction between MKRN3 and IGF2BP1 was validated by co-immunoprecipitation (co-IP). HEK293T cells were transiently transfected with equal amounts of HA-tagged human MKRN3, GFP-tagged human IGF2BP1, or both. Lysates were immunoprecipitated using anti-HA antibody. Both input and immunoprecipitated fractions were immunoblotted using anti-IGF2BP1 or anti-HA antibodies. Results showed that IGF2BP1 was co-immunoprecipitated by anti-HA antibodies when coexpressed with HA-MKRN3, verifying their protein-protein interaction (Figure 8B).

Several additional proteins that may have relevance to MKRN3’s role in pubertal initiation were identified to interact with MKRN3. WD repeat-containing protein 11, the E3 ubiquitin-protein ligase RNF114, and KISS1R have been associated with HH, with KISS1R also associated with CPP. Proteins involved in cytoskeleton pathways and proteins associated with GPCR-, DNA-, and RNA-related functions, and ubiquitin processes were also identified to interact with MKRN3.

## Discussion

The identification of MKRN3 as an inhibitor of puberty initiation opened a new door to understanding the control of puberty timing (1). The network overseeing GnRH neuronal activity has been progressively uncovered, highlighting the prominent roles of 2 activators, kisspeptin and NKB (10, 19). However, the mechanisms underlying inhibitory inputs remained poorly understood. In addition to neuroendocrine and physiological modifications, the hypothalamus undergoes a series of morphologic and synaptic changes throughout pubertal development (19, 20). In this study, we examined the contributions of MKRN3 to the neuroendocrine, molecular, and neuroplasticity changes that arise during this critical period of development.

Using hiPSCs differentiated into human ARC hypothalamic neurons, we first showed that *MKRN3* is not essential for hypothalamic differentiation but contributes to the regulation of hypothalamic development and plasticity (Figures 1 and 2). Genes important for extracellular matrix organization, axon guidance, and synapse organization pathways were significantly enriched in the *MKRN3*-KO compared with *MKRN3*-WT hypothalamic ARC neurons, including *SLIT1*, *SLIT2*, and *NRCAM* (Figure 2). These genes have been shown to be involved in the development of the hypothalamus, particularly during early postnatal development of the ARC and in the development of GnRH neurons (35, 36, 48). Hypothalamic ARC neurons derived from hiPSCs have been shown to be morphologically and molecularly similar to their in vivo counterparts (29), giving us a unique opportunity to study the actions of *MKRN3* directly in a model of the human hypothalamus.

To further explore the physiological roles of Mkrn3, we generated an *Mkrn3*-deficient mouse model. *Mkrn3* deletion was associated with an advanced age of the first estrus in female mice and a trend toward an earlier age of preputial separation in male mice (Figure 3). Female cyclicity and male and female fertility in adulthood were unaffected (Supplemental Figure 3). These results are very similar to the phenotypes in humans with *MKRN3* loss-of-function mutations; girls have advanced pubertal development, with subsequent normal fertility and menstrual periods. Boys harboring loss-of-function *MKRN3* mutations may present with definitively advanced puberty onset or a trend toward early pubertal development. It is unclear if this less severe phenotype in boys than in girls (and in male mice compared with female mice) is due to difficulty detecting early pubertal development in males in both species (1, 3). Preputial separation and testicular enlargement are less obvious than vaginal opening and breast development and may go unnoticed. Furthermore, these physical signs are “surrogates” of reactivation of the reproductive axis and are not perfect markers of reactivation of the HPG axis. While it is possible that MKRN3 may have greater effects in females than males, it is also possible that MKRN3 has similar inhibitory effects in both males and females, but we have better tools to detect these physical effects in female rodents and humans. Also, similar to humans with loss-of-function *MKRN3* mutations (3, 49), no difference in body weight was observed in our *Mkrn3*^KO^ compared to *Mkrn3*^WT^ mice. Another *Mkrn3*-deficient mouse model, generated using TALEN technology, showed similar results to our findings regarding puberty initiation, but with a more marked phenotype (i.e., significant changes in the day of female vaginal opening and male preputial separation); there is no description of cyclicity or fertility in their model (24). A notable difference between our *Mkrn3*^KO^ mouse model and the TALEN-generated *Mkrn3*-deficient mouse is the reduced body weight in their model compared with age-matched WT controls (24). Variations in strains and/or strategies for generating the mouse models may explain these differences.

We then investigated the neuroendocrine mechanisms underlying the effect of *Mkrn3* deletion on puberty initiation. We found that postnatal *Gnrh1* mRNA expression in the POA was not different in *Mkrn3*^KO^ mice compared to controls (Figure 4). These results are consistent with Yellapragada and colleagues, who showed that *MKRN3* deletion in hiPSC-derived *GNRH1*-expressing neurons does not affect *GNRH1* mRNA expression (28), although another study reported that *Mkrn3* deletion induces an increase in murine hypothalamic *Gnrh1* mRNA levels (24). Again, the discrepancy with our results may be explained in part by differences in methodologies used to generate the mouse models. At the protein level, our studies showed that *Mkrn3* deletion did not affect the number of GnRH neurons in the rPOA or the density of GnRH-ir fibers in the ME (Figure 5, A–D). In a recent mouse model of prepubertal Mkrn3 overexpression, we also found that the number of GnRH neurons in the rPOA was unchanged (50). We also examined the morphology of GnRH neurons in the rPOA at PND15 and showed no differences between *Mkrn3*^KO^ and *Mkrn3*^WT^ mice (Figure 6, A–D). Thus, although a role for Mkrn3 in synchronizing GnRH neurons’ activity cannot be excluded, Mkrn3 may exert its inhibitory influences upstream of GnRH neurons.

In parallel with the decline in *Mkrn3* expression, *Kiss1* and *Tac3* expression increase in the hypothalamus during postnatal/prepubertal development (Figure 4, B and D) (39, 40). Given our previous finding that MKRN3 associates with and represses the *KISS1* and *TAC3* gene promoters in vitro (6), we assessed the effect of *Mkrn3* deletion on kisspeptin and/or NKB levels in our *Mkrn3*^KO^ mouse and in our *MKRN3*-deficient hiPSC-derived hypothalamic ARC cells. We did not detect an effect of *Mkrn3* deletion on *Kiss1* mRNA levels in the POA (Figure 4D) or on *Kiss1* or *Tac3* mRNA levels in the ARC in our *Mkrn3*^KO^ mouse model (Figure 4, A and B), or in our *MKRN3*-deficient hypothalamic ARC cells (Figure 1, H and I, and Supplemental Figure 1, I and J). It is possible that this absence of effect is the result of compensatory mechanisms occurring in these models since *MKRN3* has been deleted from early stages of development, whereas in the previous study we showed an effect of MKRN3 on gene expression in vitro. Moreover, at any given age during the pubertal transition, in the context of the advance of pubertal onset in the absence of MKRN3, the sex steroid milieu may act to inhibit *Kiss1* and *Tac3* gene expression. Kisspeptin protein levels were also not significantly different in the RP3V and ARC in *Mkrn3*^KO^ compared to *Mkrn3*^WT^ mice. However, *Mkrn3* deletion was associated with increased NKB protein levels in the ARC at PND25 (Figure 5, N and P). These findings are corroborated by a decrease in NKB protein levels observed at PND28 in the mouse model of prepubertal Mkrn3 overexpression (50). Given that the stimulatory role of NKB on puberty initiation is well established, these findings suggest that the advanced puberty observed in the *Mkrn3*^KO^ mice may be due, at least in part, to an inhibitory action of Mkrn3 on NKB protein levels in the ARC.

Puberty is a sensitive period of maturation of the hypothalamic GnRH neural network. Findings from our in vitro study using hiPSC-derived hypothalamic ARC neurons highlighted the contribution of MKRN3 to the regulation of genes involved in extracellular matrix organization, axon guidance, and synapse organization (Figure 2). These data are further supported by the in vivo analysis of dendritic spine density in ARC neurons, where we observed a significant increase in the number of dendritic spines at PND15, from *Mkrn3*^KO^ mice compared with *Mkrn3*^WT^ controls (Figure 6, E and F). It is important to note that the Golgi staining method does not discriminate the identity of these neurons. Taken together, these findings support a significant role for Mkrn3 in the regulation of developmental plasticity in the ARC. Further investigations will allow a better understanding of the mechanisms of action of Mkrn3 in the regulation of the NKB/kisspeptin/GnRH neural developmental network during pubertal maturation (20, 39, 41).

Proteomic analyses of *Mkrn3*^WT^ and *Mkrn3*^KO^ animals at PND15 identified Igf2bp1 as a target of Mkrn3 (Figure 7). Immunohistochemical analysis confirmed that Mkrn3 reduces Igf2bp1 protein levels in the ARC at PND15. Igf2bp1 belongs to a conserved family of mRNA-binding proteins that play essential roles during embryogenesis (51). Indeed, *Igf2bp1* is highly expressed during murine early development, followed by a decline in expression around birth (52). The expression of Igf2bp1 has been shown to be negligible in adulthood, with the exception of reproductive tissues (ovaries and testes) (53). Nevertheless, modest expression has been shown in the adult male brain, and our analysis revealed that Igf2bp1 is still expressed in the ARC at PND15, indicating an important role in this hypothalamic nucleus (Figure 7) (51). Further investigation of the postnatal distribution of Igf2bp1 and its temporal colocalization with Mkrn3, kisspeptin, and NKB will be instrumental for a better understanding of the molecular mechanisms underlying puberty initiation.

Interactome analyses demonstrated direct interaction of Mkrn3 with Igf2bp1 (Figure 8). Igf2bp1 is involved in the localization and translation of mRNA targets, including *Igf2*, *Actb*, and *Pabpc1* (51, 52). Interestingly, PABPs were the proteins with the highest scores in our interactome data, indicating a high abundance of PABP proteins interacting with MKRN3 (Figure 8). Consistent with our observations, recent studies also reported that MKRN3 interacts with and ubiquitinates several members of the PABP family (26, 54). It has been shown that PABPs and IGF2BP1 are components of a heteromeric ribonucleoprotein complex required for optimal regulation of mRNA translation (47). In addition, PABPCs have been found to interact with MKRN3 to regulate *GNRH1* transcription, indicating a role in the reproductive axis (26). Thus, supported by the literature and our co-IP studies (Figure 8), our data suggest a model in which the inhibitory action of MKRN3 may involve a ribonucleoprotein complex containing MKRN3, IGF2BP1, and PABPs. IGF2BP1 has been described to be important to maintain cell adhesion and cytoskeletal integrity (55), to influence growth cone dynamics in neuronal cells (56), as well as to contribute to the development of dendritic morphology in hippocampal neurons (57). In this respect, we saw an enrichment of genes and proteins associated with cytoskeletal organization and cell adhesion in our RNA and interactome studies (Figure 2C and Figure 8A). Taken together, these data suggest important roles of MKRN3 and IGF2BP1 during hypothalamic development.

In summary, using multiple in vitro and in vivo approaches, we have shown that MKRN3 is involved in the regulation of hypothalamic development and plasticity in the ARC. We demonstrated that, similar to the human phenotype, *Mkrn3* deletion induced an advanced puberty onset in females and a trend toward an earlier age of puberty initiation in male mice, without affecting estrous cyclicity or fertility in adulthood. We further showed that while *Mkrn3* deletion did not affect hypothalamic *Gnrh1*, *Kiss1*, and *Tac3* mRNA levels, it did affect postnatal NKB protein levels in the ARC, suggesting a posttranscriptional mechanism of action. These results were complemented by the identification of a new player in the regulation of puberty initiation, IGF2BP1. During postnatal development, MKRN3 interacts with IGF2BP1 and reduces its protein levels in the ARC. The interaction of MKRN3 with PABPs supports a model in which MKRN3 actions involve the formation of a complex containing IGF2BP1 and PABPs. Taken together, these data advance the current understanding of pubertal initiation, which proposes that rather than being the result of a sudden neural network awakening after a period of quiescence, it results from a series of molecular and plastic changes that occur dynamically throughout hypothalamic development. This study reveals that MKRN3 is a major player in these processes, culminating in pubertal activation of the HPG axis.

## Methods

### Generation of MKRN3-WT and MKRN3-KO human pluripotent stem cell lines.

All hiPSC work was approved by the Brigham and Women’s Hospital IRB (protocol 2005P001440) and Institutional Biosafety Committee (protocol 2019B000117). The healthy male control hiPSC line BJ SipS-C was obtained from Harvard Stem Cell Institute (Harvard University). hiPSCs were maintained on a Matrigel-coated (Corning) plate in mTesR-1 medium (StemCell Technologies) and were passaged by enzymatic digestion using Gentle Cell Dissociation Reagent (StemCell Technologies). For CRISPR/Cas9 targeting, a genomic site in the exon of MKRN3 was subcloned into a guide RNA cloning vector (Addgene U6GRAN plasmid 68370) using Gibson Assembly Master Mix (New England Biolabs E2611S/L), 2.0 μg of Cas9 and 1.0 μg of MKRN3 guide RNA (sequence in Supplemental Table 2). Plasmids were electroporated into two 6-well plates containing 1.0 × 10^6^ cells/well dissociated BJ SipS-C hiPSCs using the Amaxa 4D-Nucleofector X Unit (Lonza). Forty-eight hours after transfection, hiPSCs were dissociated and collected by FACS. Individual clones were screened for genomic mutation by PCR amplification around the target site, followed by Sanger sequencing.

### Hypothalamic differentiation of hiPSCs.

Hypothalamic differentiation of *MKRN3*-WT and *MKRN3*-KO hiPSCs was performed as previously described (29, 58). In brief, hiPSCs were plated on Matrigel in mTeSR-1 medium containing 10 μM Y27632 (DNSK International) at a density of 100,000 cells per cm^2^. The following day (day 0), mTeSR-1 was replaced with N2B27 medium (58) containing 10 μM SB431542 (DNSK International), 100 nM LDN193189 (DNSK International) and 2 μM XAV939 (DNSK International). On day 2, 1 μM smoothed agonist (DNSK International) and 1 μm purmorphamine (DNSK International) were added to the culture medium. N2B27 medium was replaced every other day from day 2 to day 8 with changes in compound concentrations as described (58). On day 8, 5 μM DAPT (DNSK International) was added to the culture medium to promote neurogenesis, and from day 10 to day 14, the cells were maintained in N2B27 containing only 5 μM DAPT. On day 14, neural progenitors were dissociated and replated (50,000 cells per cm^2^) in N2B27 medium containing 10 ng/mL brain-derived neurotrophic factor (R&D Systems) to encourage neuronal maturation. Half of the maturation medium was changed every other day until day 30. Three separate differentiations were performed for each hiPSC clone.

### RNA extraction, reverse transcription PCR, and RT-qPCR.

Total RNA was extracted from hiPSCs, NPCs (day 15), and hypothalamic ARC neurons (day 30) using the RNeasy Mini Kit (QIAGEN) and quantified using a NanoDrop 1000 spectrophotometer (Thermo Fisher Scientific). RNA (500 ng) was reverse-transcribed using the iScript cDNA synthesis kit (Bio-Rad). Quantitative PCR assays were performed on a QuantStudio 3 Real-Time PCR system (Applied Biosystems). iQ SYBR Green Supermix (Bio-Rad) was used according to the manufacturer’s instructions. Data were normalized using *GAPDH* and *B2M* as housekeeping genes. A list of the primers used is provided in Supplemental Table 2.

### Whole transcriptome sequencing.

Total RNA was extracted from the hiPSC-derived hypothalamic ARC neurons on day 30 of maturation using the RNeasy Mini Kit. RNA from 3 separate differentiations were extracted for *MKRN3*-WT 1 and *MKRN3*-KO 1 hypothalamic neurons. Sample mRNA libraries were prepared using the Illumina Tru-Seq Stranded mRNA kit and sequenced on an Illumina HiSeq 4000 system with a paired-end run and a depth of 50 million aligned reads, by the Broad Institute Genomics Platform. RNA-Seq analysis was done by the Harvard Chan Bioinformatics Core. RNA-Seq counts were generated by bcbio and bcbioRNASeq using salmon. Counts were imported into R using tximport, and DEGs were analyzed with the R package DESeq2. DEGs were identified using the Wald test. Gene annotations were obtained from Ensembl. Differential expression analysis was performed using a BH-adjusted *P* value cutoff of 0.05 and a log fold-change (LFC) ratio cutoff of 1. To identify the main functions of DEGs, enriched GO terms were identified using the g:Profiler toolkit. DEGs that met both criteria of adjusted *P* < 0.05 and LFC > 1 were used as input for GO analysis. The RNA-Seq data supporting this publication are available in the Gene Expression Omnibus repository under GSE208722.

### Mice.

All experiments were performed in agreement with the NIH and the IACUC at Brigham and Women’s Hospital. Mice were group-housed and maintained in a 12-hour light/12-hour dark cycle, under constant conditions of temperature (22°C–24°C) and ad libitum access to standard mouse chow and water.

*Mkrn3*-KO mice were generated from embryonic stem cells carrying a deletion of the *Mkrn3* gene (Chr7:62,420,038-62,418,286) and an insertion of a *LacZ* marker, obtained from the Knockout Mouse Project repository (project VG11253). Blastocyst injections (on a C57BL/6 albino background) yielded chimeric male mice that upon crossing to WT C57BL/6 female mice were confirmed to carry the *Mkrn3* deletion in the germline. *Mkrn3* is maternally imprinted and hence expressed only from the paternal allele in both mice and humans (4, 5). By convention, the maternal allele is shown first, so *Mkrn3*^+/–^ mice have inherited the mutant allele from their father, while *Mkrn3*^–/+^ mice have inherited the mutant allele from their mother. We first generated *Mkrn3*^+/–^ and *Mkrn3*^–/+^ mice by crossing *Mkrn3*-KO (*Mkrn3*^+/–^) males with *Mkrn3*^+/+^ females or *Mkrn3*-KO (*Mkrn3*^+/–^) females with *Mkrn3*^+/+^ males, respectively. The analysis of *Mkrn3* expression in the hypothalamus showed expression of *Mkrn3* in *Mkrn3*^–/+^ mice, whereas it was absent in *Mkrn3*^+/–^ mice that carried the mutation from their father (Supplemental Figure 2). These results confirmed the imprinting of *Mkrn3* in this mouse model. Subsequent analyses compared *Mkrn3*^+/–^ (named *Mkrn3*^KO^) and *Mkrn3*^+/+^ (named *Mkrn3*^WT^) littermates in a 129S1/SvlmJ genetic background, generated by crossing *Mkrn3*^KO^ males with *Mkrn3*^WT^ females. Genotyping was performed by PCR using DNA isolated from ear biopsies, using primers to recognize the presence of the *Mkrn3* WT allele, and the insertion of the *LacZ* marker (primers detailed in Supplemental Table 2).

### Pubertal and adult reproductive physiology.

Female mice were examined daily for evidence of vaginal opening and first estrus from weaning. Vaginal smears were taken daily from the day of vaginal opening to adulthood to determine the day of first estrus and the following estrous cyclicity of adult females. The estrous cycle stage was determined by microscopy after hematoxylin-eosin coloration of the vaginal smears. Male mice were monitored daily from weaning for preputial separation, an indirect marker of puberty initiation. To evaluate the capacity of the animals to produce offspring, *Mkrn3*^WT^ and *Mkrn3*^KO^ male and female mice were individually housed with WT age-matched females or males respectively, for 4 months. The time to first litter, number of litters, and litter size were monitored. Body weight was measured weekly from weaning to adulthood.

### Mouse tissue collection for measurement of mRNA levels.

Brains of females at PND10, PND15, PND20, and PND25 were extracted and rapidly embedded in Tissue-Tek O.C.T compound (Sakura Finetek), frozen in a –50°C isopentane solution (Thermo Fisher Scientific), and stored at –80°C until use. Frozen tissue punches were extracted through the POA and the ARC with a 1 mm diameter canula (Cadence Science) as previously described (59). Total RNA was extracted using the RNeasy Micro Kit (Qiagen), then reverse-transcribed into cDNA, and RT-qPCR analysis was performed as described above. Data were normalized using *L19* and *Hprt* as housekeeping genes. A list of the primers used is provided in Supplemental Table 1.

### Immunohistochemistry and immunofluorescence.

Females at PND10, PND15, and PND25 were terminally anesthetized and transcardially perfused with a solution of 4% paraformaldehyde (PFA, Boston BioProducts) in phosphate buffer (PB). Brains were postfixed overnight in PFA-PB, cryoprotected in 20% sucrose-PB (Thermo Fisher Scientific), and stored until analyses, as previously described (60). Brains were sliced into 30 μm coronal sections using a freezing stage microtome (Microm HM440E). Free-floating sections were blocked for 2 hours with 2% normal goat serum (Sigma-Aldrich) in PBS-Triton 0.3%, then incubated with rabbit anti-Mkrn3 (1:500, Sigma HPA029494), rabbit anti-GnRH (1:1,000, Immunostar LHRH 20075), or rabbit anti-Igf2bp1 (1:5,000, MBL RN007P) antibody for 24 hours, or either rabbit anti-kisspeptin (1:2,000, INRAE AC566) or rabbit anti–neurokinin B (1:1,000, Novus Biologicals NB300-201) antibody for 48 hours at 4°C. Mkrn3 immunostaining and GnRH immunostaining in the rPOA were performed for 2 hours at room temperature using a biotinylated goat anti-rabbit antibody (1:500, Vector Laboratories BA-1000), followed by a 1-hour incubation with the Vectastain Elite ABC avidin/biotin-based peroxidase system (Vector Laboratories) and color development with the Vector DAB substrate kit or DAB/Nickel substrate kit, respectively (Vector Laboratories). GnRH, kisspeptin, NKB, and Igf2bp1 immunofluorescence stainings were performed for 2 hours at room temperature using a goat anti-rabbit DyLight 488 secondary antibody (1:500, Thermo Fisher Scientific 35553). Brain sections were dried and mounted with DPX mountant (Sigma) for immunostaining or Vectashield antifade mounting medium containing DAPI for immunofluorescence (Vector Laboratories).

### Microscopy and image analysis.

Mkrn3 expression image analysis was performed using a Nikon Eclipse 90i microscope. Anatomically matched sections were selected at the level of the POA (plates 29 of the Mouse Brain Atlas of Paxinos and Franklin) and ARC (plates 45) and compared between *Mkrn3*^WT^ and *Mkrn3*^KO^ mice. Subsequent image analysis was performed using a ZEISS LSM 710 confocal microscope. GnRH-immunoreactive neurons were examined on 3 anatomically matched sections selected at the level of the rPOA (plates 26–27). GnRH neurons were scored as being unipolar, bipolar, or complex according to their dendritic tree structure as previously described (21). GnRH fiber density at the level of the ME (plate 45, 2 sections per animal) was quantified by voxel counts on a set of 30 serial image planes (*z* step distance = 0.83 μm). The number of kisspeptin cells was counted in the AVPV/PeN (plates 28 to 30, 2 sections per animal). Analysis of total kisspeptin, NKB, and Igf2bp1 immunoreactivity was carried out in the medial region of the ARC (plate 45, 2 sections per animal) by voxel counts on a set of 30 serial image planes (*z* step distance = 0.83 μm).

### Golgi staining.

Female mice were sacrificed at PND15, and the brains were immersed in Golgi-Cox solution for 2 weeks (FD Rapid GolgiStain Kit, FD Neurotechnologies). Coronal sections (150 μm) were cut on a cryostat (Leica, CM3050 S) and mounted on gelatin-coated microscope slides (FD Neurotechnologies). Sections containing the ARC were immersed in postimpregnation solution for 10 minutes, washed in milli-Q water (MilliporeSigma), dehydrated, and coverslipped with Permount mounting medium (Thermo Fisher Scientific). Light microscopy imaging and stacks of serial optical sections were acquired with a ZEISS Axioskop 2 Plus microscope using a ×100 objective. Ten neurons distributed throughout the ARC were analyzed for each animal. The number of dendritic spines was counted within a 50 μm segment, situated at 50 μm from the soma of the neuron. Image analysis and quantification of the spines were performed using ImageJ (NIH) and NeuronStudio software.

### Hypothalamic protein extraction and Western blot analysis.

Female mice were sacrificed at PND10, and brains were removed from the skull. The caudal area of the hypothalamus that include the MBH was isolated as previously described (6) and stored at –80°C until use. Total protein was extracted from frozen MBH using RIPA lysis buffer system supplemented with protease inhibitors (Santa Cruz Biotechnology) for 30 minutes on ice, and then the samples were centrifuged for 15 minutes at 20,000*g* at 4°C. Protein concentrations were determined by BSA protein assay (Thermo Fisher Scientific). Equal amounts of protein were separated by SDS-PAGE on 10% polyacrylamide gels, electro-transferred onto nitrocellulose membranes (Bio-Rad), blocked for 1 hour with 5% milk in TBS-Tween 0.1%, and incubated with rabbit anti-Mkrn3 antibody (1:500, Abcam, ab140267) overnight at 4°C. The blots were then incubated with an anti-rabbit HRP-conjugated secondary antibody (1:5,000, Bio-Rad 170-6515), and protein detection was done using the HyGlo chemiluminescence HRP antibody detection reagent (Denville Scientific). Protein levels were normalized to β-actin.

### TMT-proteomics.

Brains of mice at PND15 were extracted, rapidly embedded in Tissue-Tek O.C.T compound (Sakura Finetek), and frozen in a –50°C isopentane solution (Thermo Fisher Scientific). Frozen tissue punches were extracted through the ARC with a 1 mm diameter cannula as described above and stored at –80°C until use. Samples were processed using the streamlined TMT labeling protocol (45). Mass spectra were collected on Orbitrap Fusion mass spectrometer (Thermo Fisher Scientific) coupled to a Proxeon EASY-nLC 1200 LC pump (Thermo Fisher Scientific) using an SPS-MS3 method as previously described (45). Mass spectra were processed using a COMET-based software pipeline, searched against the UniProt Mouse database (April 2019), and quantified as previously described (45). Additional details are available in the Supplemental Methods. Quantified proteins are available in Supplemental Table 3, and all data have been deposited to the ProteomeXchange Consortium via the PRIDE partner repository with the data set identifier PXD027220 (61).

### Cell culture of HEK293T cells.

The HEK293T cell line (ATCC) was cultured in DMEM (MilliporeSigma) supplemented with 10% FBS (Invitrogen) and 1% penicillin/streptomycin solution (Invitrogen). Cell growth medium was warmed before contact with cells. Cells were incubated in a humidified incubator at 37°C and 5% CO_2_.

### Interactome.

HEK293 and SHSY-5Y cells (ATCC) were plated in 15 cm cell culture plates and were transiently transfected using Fugene with a plasmid encoding MKRN3 with N-terminal or C-terminal FLAG and HA tags (phage-N-FLAG-HA and phage-C-FLAG-IRES_PURO). Stable HEK293 cells were also generated by supplementation of the standard growth media (high-glucose DMEM + 10% FBS) with 1 μg/mL puromycin. For protein purification, cells from four 15 cm tissue culture dishes at approximately 80% confluence were lysed in a total volume of 4 mL of mammalian cell lysis buffer (MCLB Abcam 179835) with Roche complete EDTA-free protease inhibitor cocktail for 1 hour with gentle rocking at 4°C. Lysates were cleared using centrifugation (20,000*g* at room temperature, 10 minutes), the supernatant was filtered through 0.45 μm spin filters (MilliporeSigma) to further remove cell debris, and the resulting material was subjected to IP with 60 μL of immobilized anti-HA (Sigma) resin (50% slurry) overnight at 4°C with gentle inversion. Resin containing immune complexes was washed with 1 mL ice cold lysis buffer 8 times followed by three 1 mL PBS washes. Proteins were eluted with three 50 μL incubations with 250 μg/mL HA-peptide (Sigma) in PBS for 30 minutes each at 22°C, and elutions were pooled for a final volume of 150 μL. Proteins in each elution were precipitated with 20% trichloroacetic acid (TCA), and the resulting pellet was washed once with 10% TCA and 3 times with cold acetone.

### MS.

TCA-precipitated proteins were trypsinized, purified using Empore C18 extraction media (3M), and analyzed via liquid chromatography-tandem mass spectrometry with an LTQ linear ion trap mass spectrometer (Thermo Finnigan, Thermo Fisher Scientific) using an 18 cm × 125 μm (inner diameter) C18 column and a 50-minute 8%–26% acetonitrile gradient. Spectra were searched using Sequest against a target-decoy human tryptic peptide database, and these results were loaded into CompPASS for further processing and analysis (46).

### Co-IP.

Human MKRN3 (3×HA-tagged) pReceiver plasmid was purchased from GeneCopoeia (EX-F0470-M06). Human IGF2BP1 (GFP-tagged) mammalian expression vector was purchased from OriGene (RG216226). HEK293T cells were plated at a density of 0.3 × 10^6^ cells/well. After 24 hours, cells were transiently transfected with either 0.5 μg/well HA-MKRN3, 0.5 μg/well of GFP-IGF2BP1, or 0.5 μg/well of HA-MKRN3 and 0.5 μg/well of GFP-IGF2BP1, using Lipofectamine 2000 (Invitrogen). The total amount of DNA transfected was kept constant at 1 μg/well by adding the appropriate amount of empty vector. Twenty-four hours after transfection, cells were harvested and lysed in IP Lysis buffer (Thermo Fisher Scientific) supplemented with protease inhibitor (Roche Diagnostics Ltd.). We retained 10% of the protein for a total lysate sample (input). The remainder of the samples were incubated with rabbit anti-HA (Abcam, ab9110) overnight at 4°C. Antibody-antigen complexes were precipitated with protein A beads (Invitrogen) for 3 hours at room temperature and washed 3 times before elution with 5× SDS-PAGE sample buffer and detection by Western blot; proteins were separated by SDS-PAGE and transferred onto a nitrocellulose membrane (Promega). After blocking with 5% nonfat milk in TBS containing 0.1% Tween 20 for 1 hour at room temperature, membranes were incubated overnight at 4°C with mouse anti-IGF2BP1 (Medical & Biological Laboratories Co., Ltd., RN001M, diluted 1:1,000). The membranes were then probed for 1 hour with anti-mouse secondary antibody conjugated with HRP (Bio-Rad, 1706515, diluted 1:10,000). Bands were detected using an enhanced chemiluminescence reagent (PerkinElmer) and images acquired with ChemiDoc XRS+ (Bio-Rad).

### Statistics.

All data are expressed as mean ± SEM. Two-tailed *t* test was used to analyze the variance between 2 groups. Two-way ANOVA, followed by Tukey’s post hoc test, was used to determine significance of experiments comparing multiple factors. Significance level was set at *P* value less than 0.05. All analyses were performed with GraphPad Prism software.

### Study approval.

All mouse studies were approved by the IACUC of Brigham and Women’s Hospital. All animal experiments were carried out in accordance with the NIH *Guide for the Care and Use of Laboratory Animals* (National Academies Press, 2011) and abided by the declaration of ethical approval for experiments.

## Author contributions

LN, AM, SAP, BMG, JRL, HKK, JL, CM, and APA performed the experiments. LN, SPG, FTM, RSC, APA, and UBK designed the experiments. LN, BMG, JLR, JCM, and APA analyzed the data. LN, APA, and UBK wrote the manuscript. SPG, RSC, APA, and UBK supervised the research data. LN, RSC, APA, and UBK conceived the research study. The manuscript was reviewed and edited by all authors.

## Supplementary Material

Supplemental data

Supplemental table 3

Supplemental table 4

## Version 1 04/24/2023

Electronic publication
