# Supplementary material for: MKRN3 inhibits puberty onset via interaction with IGF2BP1 and regulation of hypothalamic plasticity
Source: JCI Insight. 2023 Apr 24;8(8):e164178. doi: 10.1172/jci.insight.164178 (PMC10243807; doi:10.1172/jci.insight.164178)
Supplement: Supplemental data [file jciinsight-8-164178-s091.pdf]

Supplemental methods

TMT-proteomics

Brains of mice at PND15 were extracted, rapidly embedded in Tissue-Tek O.C.T compound (Sakura Finetek) and frozen in a -50˚C isopentane solution (Fisher Scientific). Frozen tissue punches were extracted through the ARC with a 1 mm diameter cannula as described above and stored at -80˚C until use. Samples were processed using the streamlined TMT labelling protocol (1). Samples were lysed in 8 M urea in 200 mM EPPS pH 8.5 with protease (Pierce A32953) and phosphatase (Pierce A32957) inhibitors and passed through a 21-gauge needle 10x. Samples were reduced with 5 mM TCEP, alkylated with 10 mM iodoacetamide, and quenched with 5 mM DTT, followed by methanol/chloroform precipitation of 100 µg. Pellets were reconstituted in 200 mM EPPS pH 8.5, digested overnight with LysC (Wako 129-02541) at 1:100 while shaking at room temperature, followed by digestion with trypsin (Pierce 90305) at 1:100 while shaking at 37°C. Anhydrous acetonitrile (Honeywell AS017-0100) was added to ~30%, followed by labelling with TMT11 (Thermo A37727) reagent. 1% of each labeled sample was combined and analyzed unfractionated to ensure labeling efficiency was >97% and that the samples are mixed at a 1:1 (total amount) ratio across all conditions. After mixing, labelled peptide samples were de-salted using a 100 mg Sep-Pak cartridge (Waters WAT054925), followed by drying in a rotary evaporator. Samples were then reconstituted in 5% ACN 10 mM ammonium bicarbonate for basic reverse phase fractionation on an Agilent 300extend-C18 column (3.5µm, 4.6x250mm) using an Agilent Infinity 1260 HPLC. Peptides were subjected to a 75 min linear gradient from 13% to 42% of Buffer B (10 mM ammonium bicarbonate, 90% ACN, pH 8) at a flow rate of 0.6 mL/min, resulting in a total of 96 fractions which were consolidated into 24 by combining (in a chessboard

pattern) four alternating wells down columns of the 96-well plate. Assuming adjacent fractions contain overlapping peaks, only 12 non-adjacent samples were analyzed by the mass spectrometer. The pooling scheme has been illustrated previously (1, 2). Each eluted fraction was desalted via StageTip for SPS-MS3 analysis.

Mass spectra were collected on Orbitrap Fusion mass spectrometer (ThermoFisher Scientific) coupled to a Proxeon EASY-nLC 1200 LC pump (ThermoFisher Scientific). Peptides were separated on a 35 cm column (i.d. 100 μm, Accucore, 2.6 μm, 150 Å) packed in-house using a 150 min gradient (from 5% -30% acetonitrile with 0.1% formic acid) at 500 nl/min. Each analysis used an SPS-MS3-based TMT method (3, 4). MS1 data were collected using the Orbitrap (120,000 resolution; maximum injection time 50 ms; AGC 4e5, 400-1400 m/z). Determined charge states between 2 and 5 were required for sequencing and a 90 s dynamic exclusion window was used. MS2 scans consisted of collision-induced dissociation (CID), quadrupole ion trap analysis, automatic gain control (AGC) 2E4, NCE (normalized collision energy) 34, q-value 0.25, maximum injection time 35 ms, and isolation window of 0.7 Da using a Top10 method.

Mass spectra were processed using a COMET-based software pipeline. Data were searched against the UniProt Mouse database (April 2019), using a 20-ppm precursor ion tolerance for total protein-level analysis, 1.0005 Da product ion tolerance, and 0.4 Da fragment bin offset. TMT tags on lysine residues and peptide N termini (11-plex: +229.163 Da) and carbamidomethylation of cysteine residues (+57.021 Da) were set as static modifications, while oxidation of methionine residues (+15.995 Da), and deamidation of N and Q (-0.984 Da) were set as variable modifications. Peptide-spectrum matches (PSMs) were identified, quantified, and filtered to a 1% peptide false discovery rate (FDR) and then collapsed further to a final protein-level FDR of 1%. Proteins were quantified by summing reporter ion counts across all matching PSMs. Briefly, a 0.003 Da (3

millidalton) window around the theoretical m/z of each reporter ion was scanned and the maximum intensity nearest the theoretical m/z was used. Reporter ion intensities were adjusted to correct for the isotopic impurities of the different TMT reagents according to manufacturer specifications and adjusted to normalize ratios across labelling channels. Lastly, for each protein, signal-to-noise (S:N) measurements of the peptides were summed and then normalized to 100. The mass spectrometry proteomics data are available in Suppl. Table 3. They also have been deposited to the ProteomeXchange Consortium via the PRIDE partner repository with the dataset identifier PXD027220 (5).

1. Navarrete-Perea J, Yu Q, Gygi SP, Paulo JA. Streamlined Tandem Mass Tag (SL-TMT) Protocol: An Efficient Strategy for Quantitative (Phospho)proteome Profiling Using Tandem Mass Tag-Synchronous Precursor Selection-MS3. *J. Proteome Res.* 2018;17(6):2226–2236.

2. Paulo JA et al. Quantitative mass spectrometry-based multiplexing compares the abundance of 5000 S. cerevisiae proteins across 10 carbon sources. *J. Proteomics* 2016;148:85–93.

3. McAlister GC et al. MultiNotch MS3 enables accurate, sensitive, and multiplexed detection of differential expression across cancer cell line proteomes. *Anal. Chem.* 2014;86(14):7150–7158.

4. Ting L, Rad R, Gygi SP, Haas W. MS3 eliminates ratio distortion in isobaric multiplexed quantitative proteomics. *Nat. Methods* 2011;8(11):937–940.

5. Perez-Riverol Y et al. The PRIDE database and related tools and resources in 2019: improving support for quantification data. *Nucleic Acids Res.* 2019;47(D1):D442–D450.

Supplementary figures

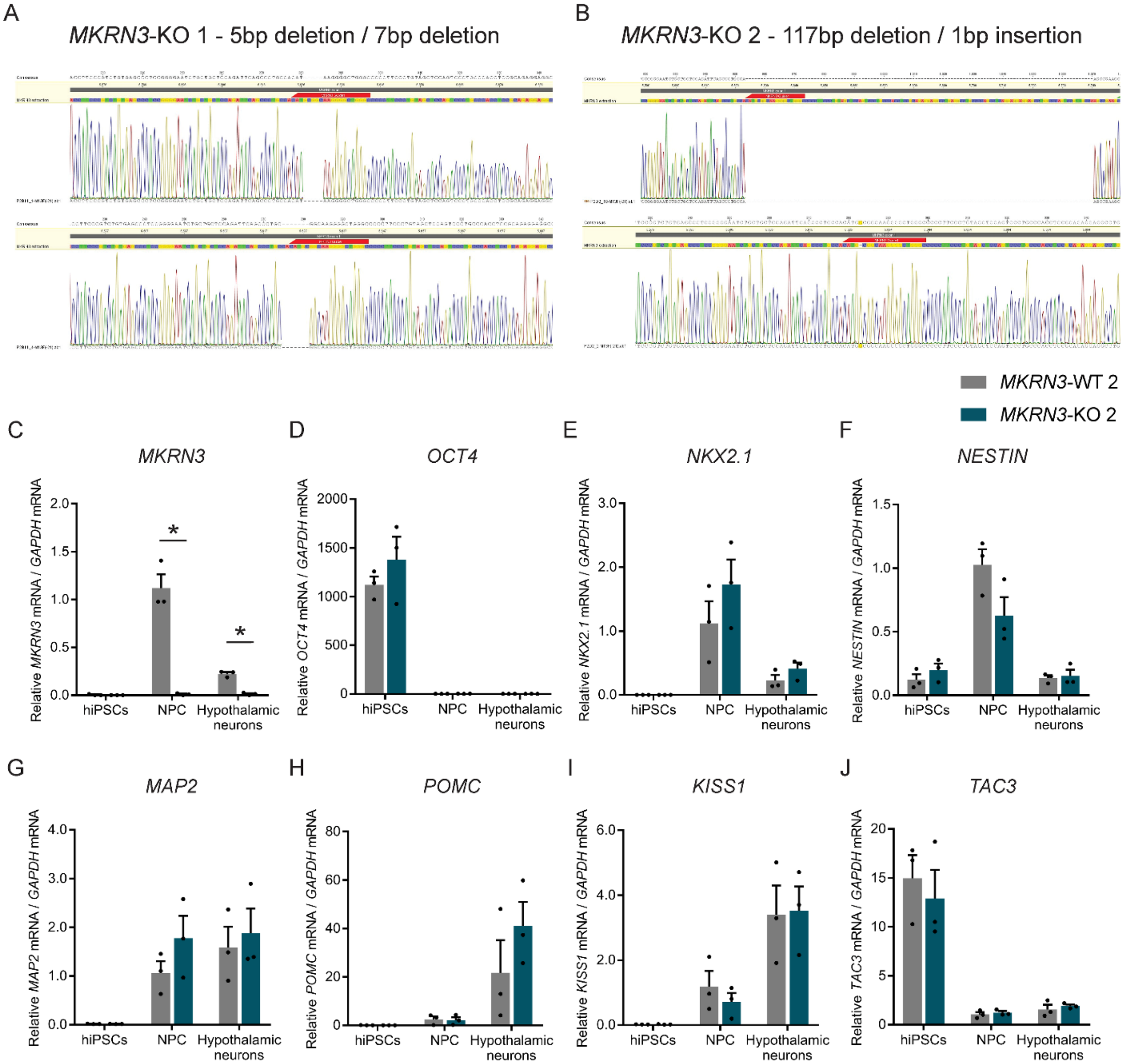

Supplementary figure 1. Generation of *MKRN3*-WT and *MKRN3*-KO hiPSC-derived hypothalamic ARC neurons. (A, B) Sanger sequencing of the two bi-allelic *MKRN3*-KO hiPSC clones showing the introduction of (A) a 5 bp deletion and a 7 bp deletion in the *MKRN3*-KO 1 and (B) a 117 bp deletion and a 1 bp insertion in the *MKRN3*-KO 2 hiPSCs, using CRISPR/Cas9 technology. (C-J) *MKRN3* (C), *OCT4* (D), *NKX2.1* (E), *NESTIN* (F), *MAP2* (G), *POMC* (H), *KISS1* (I) and *TAC3* (J) mRNA levels in hiPSCs, NPCs and hypothalamic ARC neurons derived from the *MKRN3*-WT 2 and *MKRN3*-KO 2 clones (n = 3 differentiation protocols per group), relative to levels in *MKRN3*-WT 2 NPCs. Data are presented as the mean ± SEM values. Statistics were performed using two-way ANOVA, followed by Tukey post-hoc test. *$p < 0.01$.

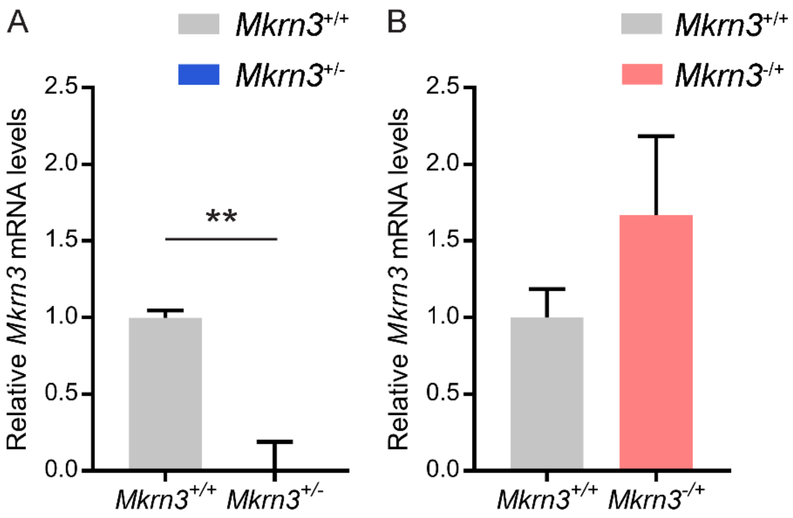

Supplementary figure 2. *Mkrn3* is maternally imprinted and expressed only from the paternal allele in the mouse hypothalamus. (A) Relative *Mkrn3* mRNA levels in the hypothalamus of *Mkrn3*$^{+/+}$ (wild-type) and *Mkrn3*$^{+/-}$ (deletion originated from the paternal allele) mice at postnatal day 10 (n = 3 per genotype). (B) Relative *Mkrn3* mRNA levels in the hypothalamus of *Mkrn3*$^{+/+}$ (wild-type) and *Mkrn3*$^{-/+}$ (deletion originated from the maternal allele) mice at postnatal day 10 (n = 3-4 per genotype). Statistics were performed using unpaired two-tailed *t*-test. Data are presented as the mean ± SEM values. **$p < 0.01$ compared to *Mkrn3*$^{+/+}$.

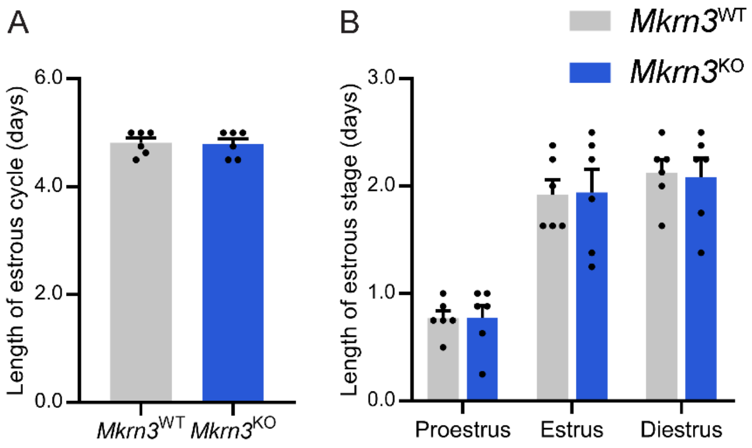

Supplementary figure 3. *Mkrn3* deletion does not modify adult female cyclicity. (A) Average length of the estrous cycle in *Mkrn3*^{KO} females compared to *Mkrn3*^{WT} (n = 6 per genotype). Statistics were performed using unpaired two-tailed *t*-test ($t_{(10)} = 0.161$, $p = 0.875$). (B) Average amount of time spent in each stage of the estrous cycle in *Mkrn3*^{WT} and *Mkrn3*^{KO} females (n = 6 per genotype). Statistics were performed using two-way ANOVA, followed by Tukey post-hoc test (genotype effect: $F_{(1,30)} = 0.002$, $p = 0.956$). Data are presented as the mean ± SEM values.

Supplementary Tables

Supplementary Table 1. Fertility analysis of *Mkrn3*^{WT} and *Mkrn3*^{KO} mice.

	Female		Male	
	Mkrn3^{WT}	*Mkrn3*^{KO}	*Mkrn3*^{WT}	*Mkrn3*^{KO}
Time to first litter (days)	23.3 ± 0.3	26.3 ± 1.9	23.3 ± 0.3	26.3 ± 3.3
Number of litters	3.7 ± 0.3	4.0 ± 0.6	3.7 ± 0.3	4.0 ± 0.0
Number of pups per litter	5.00 ± 0.3	5.6 ± 0.6	5.00 ± 0.3	5.00 ± 0.5
Total number of pups	18.3 ± 2.3	20.3 ± 1.5	18.3 ± 2.3	19.7 ± 1.86

During 4 months of continuous mating, *Mkrn3*^{WT} and *Mkrn3*^{KO} males and females produced offspring with the same time to first litter (Female: $t_{(4)} = 1.591$, $p = 0.187$; Male: $t_{(4)} = 0.896$, $p = 0.421$), comparable numbers of litters (Female: $t_{(4)} = 0.500$, $p = 0.643$; Male: $t_{(4)} = 1.000$, $p = 0.374$), and comparable litter size (Female: $t_{(4)} = 0.977$, $p = 0.384$; Male: $t_{(4)} = 0.106$, $p = 0.921$), as well as total number of pups (Female: $t_{(4)} = 0.728$, $p = 0.507$; Male: $t_{(4)} = 0.447$, $p = 0.678$). Statistics were performed using unpaired two-tailed *t*-test.

Supplementary Table 2. Primers used for RT-qPCR and PCR.

Gene	NCBI Reference Sequence	Forward primer	Reverse primer
CRISPR-Cas9 targeting			
MKRN3 gRNA	--	TTTCTTGGCTTTATATATCTTGTGGAA<br>AGGACGAAACACCGCCCAGCCCCTTG<br>CCGCATG	GACTAGCCTTATTTTAACTTGCTATTT<br>CTAGCTCTAAAACCATGCGGCAAGGG<br>GCTGGGC
RT-qPCR - Human			
MKRN3 del1	NM_005664.4	ATTCAGCCCTGCCACATGCG	CTTCGGCTTGGCAACGGAC
MKRN3 del2	NM_005664.4	ATTCAGCCCTGCCACATGCG	GTCGTGCGAATAGCGACAGT
OCT4	NM_002701.6	GGTTCTATTTGGGAAGGTATTCAG	GGTTCGCTTTCTCTTTCGG
NKX2-1	NM_001079668.3	CAGGACACCATGAGGAACAGCG	GCCATGTTCTTGCTCACGTCCC
NESTIN	NM_006617.2	GGCGCACCTCAAGATGTCC	CTTGGGGTCCTGAAAGCTG
MAP2	NM_002374.4	TAACCAACCACTGCCAGACCTGAA	GCCACATTTGGATGTCACATGGCT
POMC	NM_001035256.3	CTGGAGAGCAGCCAGTGTCAG	AGAGGCTGCTCGTCGCCATTTC
KISS1	NM_002256.4	GCACTTCTAGGACCTGCCTC	GATTCTAGCTGCTGGCCTGTG
TAC3	NM_013251.4	CAAAAGCCACTCATCTCTGGAGG	GCTCCTCTTGCCCATAAGTCCC
SLIT1	NM_003061.3	CTCCTTCACCAACATGAGCCAG	AGGGTGGAGATGTCATTGCCGT
SLIT2	NM_004787.4	CAGAGCTTCAGCAACATGACCC	GAAAGCACCTTCAGGCACAACAG
NRCAM	NM_001037132.4	TGTGGCTGAAGGACAACAGGGA	AGACGCTGTCCAGAGTGGTGTT
RT-qPCR - Mouse			
Mkrn3	NM_011746.3	AAGCGCATACTGGCATCAAG	AGCCAACGGTCATCAGAGAA
Kiss1	NM_178260.3	GCTGCTGCTTCTCCTCTGTG	TCTGCATACCGCGATTCCTT
Kiss1r	NM_053244.5	GGTGCTGGGAGACTTCATGT	ACATACCAGCGGTCCACACT
Gnrh	NM_008145.3	GGGAAAGAGAAACACTGAACAC	GGACAGTACATTCGAAGTGCT
Tac3	NM_009312.2	GCTCCACAGCTTTGTCCTTC	GCTAGCCTTGCTCAGCACTT
Tac1	NM_009311.3	ACCAGATCAAGGAGGCAATG	AGCCTTTAACAGGGCCACTT
PCR - Mouse			
Mkrn3-WT		GCCATATGCTCTCTCTCAATTGCCG	AATTTGTTCCTGGACAGCCTTACCG
LacZ marker		ACTTGCTTTAAAAAACCTCCCACA	ACATGTAAACAAACACAGACAGCAGC
